# A low-cost arduino-based pulsed constant-current platform for electrochemical nanostructure synthesis

**DOI:** 10.1016/j.ohx.2026.e00831

**Published:** 2026-08-22

**Authors:** Marcos Luna Cervantes, Leandro García González, Julián Hernández Torres, Erick Octavio Santos Santiago, José Luis Zamora Navarro, Diana Jiménez Girón, Daniel de Jesús Araujo Pérez, Jorge Bertin Santaella González, Mario Alberto Díaz Solís, Pablo Thomas Dupont, Irma Yadira Izaguirre Hernández, Antonio de Jesús García Chávez

**Affiliations:** a Centro de Investigación en Micro y Nanotecnología, Universidad Veracruzana, Av. Adolfo Ruiz Cortines 455, col. Costa Verde, 94294 Boca del Río, Mexico; b Facultad de Ciencias Químicas, Universidad Veracruzana, Av. Adolfo Ruiz Cortines 455, col. Costa Verde, 94294 Boca del Río, Mexico; c Instituto de Investigaciones Médico Biológicas, Universidad Veracruzana, Iturbide s/n entre Carmen Serdán y 20 de Noviembre, 91700 Veracruz, Mexico; d Facultad de Bioanálisis, Universidad Veracruzana, Iturbide s/n Esquina Carmen Serdán, 91700 Veracruz, Mexico; e Doctorado en Ciencias e Ingeniería, Universidad Autónoma de Baja California, Carretera Transpeninsular Ensenada, Tijuana 3917, col. Playitas, 22860 Ensenada, Mexico

**Keywords:** Pulsed Current Electrodeposition, Arduino-Based, Nanostructures, SERS

## Abstract

Pulsed-current electrodeposition is widely used to control nucleation and growth during the electrochemical synthesis of metallic nanostructures; however, reproducible implementation commonly requires programmable potentiostats or galvanostats, which can be costly or inaccessible for parallel experimentation. This study introduces a low-cost Arduino-based pulsed constant-current platform designed for low-voltage electrodeposition. The system incorporates a 12-bit MCP4725 digital-to-analog converter to define the current setpoint, while an MCP602 operational amplifier drives an IRLZ34N MOSFET in a low-side feedback configuration using a 10 Ω shunt resistor. A 2N2222 transistor actively clamps the MOSFET gate during OFF periods to minimize residual conduction between pulses. Standalone firmware enables direct configuration of ON time, OFF time, cycle number, and current through a 20 × 4 IC LCD and three push buttons, without requiring an external computer during operation. Electrical evaluation from 0.50 to 40.00 mA using a test LED load showed a highly linear relationship between programmed and multimeter-indicated current, with R^2^ = 0.9998 and a maximum observed difference of 0.80 mA. Pulse timing and OFF-state behavior were confirmed by oscilloscope monitoring. Beyond electrical testing, the platform was validated through Ag electrodeposition protocols used to fabricate TiO_2_/Ag SERS substrates, including Ag nanoparticles and 3D Ag dendrites, with analytical performance within the range reported for established SERS architectures. The proposed hardware offers an accessible, reproducible, and open-source solution for laboratories requiring programmable pulsed-current capability for low-voltage electrochemical nanostructure synthesis.

## Graphical abstract

**Figure:**
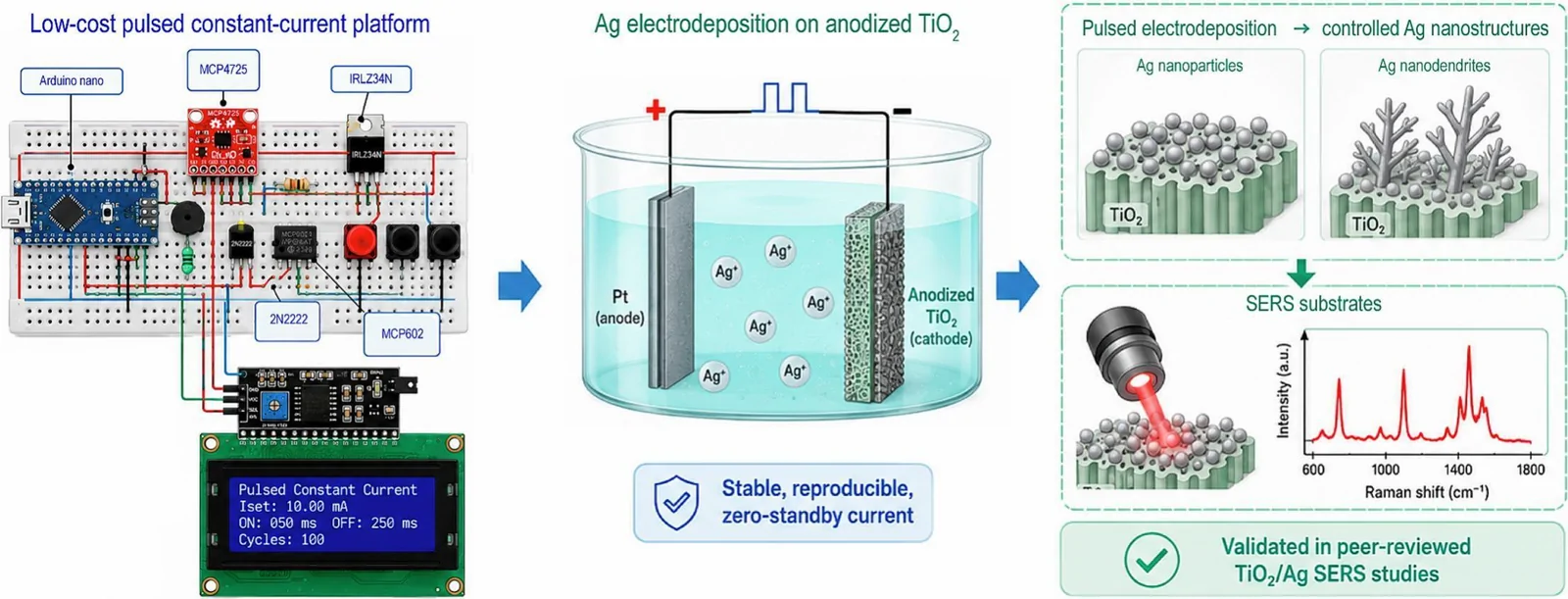

## 1 Hardware in context

Pulsed current electrodeposition (PCE) is an electrochemical synthesis strategy in which current pulses are alternated with relaxation intervals to control nucleation, growth, metal loading, spatial distribution, and final deposit morphology [1], [2], [3], [4]. During the ON interval, electrochemical reduction and metal growth occur at the electrode surface, whereas the OFF interval allows partial recovery of the diffusion layer and redistribution of electroactive species. Consequently, current or current density, ON time, OFF time, duty cycle, and cycle number can be adjusted to produce different structures, including dispersed nanoparticles, dense metallic deposits, aggregates, and branched or dendritic architectures. This control is relevant to the fabrication of functional nanostructures and electrode coatings for applications including photoelectrochemical systems, energy-related electrodes, catalytically active surfaces, and surface-enhanced Raman scattering (SERS) substrates [5], [6], [7], [8], [9], [10].

These synthesis requirements establish several practical criteria for a PCE instrument. The system must provide stable constant-current regulation within its voltage-compliance range, programmable ON and OFF intervals, reproducible cycle counting, and a well-defined zero-current state during the relaxation period. At low current levels, performance also depends on the current-sensing resistance, digital-to-analog converter resolution, supply voltage, wiring resistance, feedback-loop stability, and switching behavior of the power stage. Residual conduction during the OFF interval can modify ionic redistribution near the electrode surface and thereby affect subsequent nucleation and growth. Accordingly, the electrical behavior of the current-regulation and OFF-state circuits should be documented under representative operating conditions.

Commercial potentiostats and galvanostats offer advanced waveform control, high accuracy, and broad voltage and current ranges; however, their cost can limit the number of instruments available for parallel experimentation [11]. Open-source systems such as PassStat and MYSTAT provide lower-cost alternatives for a range of electrochemical measurements [12], [13]. The platform presented here is complementary to these instruments and was designed specifically for standalone, low-voltage pulsed constant-current electrodeposition. A dedicated system of this type can support synthesis workflows in which pulse duration, relaxation time, cycle number, and current must be varied systematically without requiring a computer during operation.

Arduino-based open hardware provides a practical basis for specialized laboratory instruments when accessible components, complete construction documentation, modifiable firmware, and validation under realistic operating conditions are provided [14], [15], [16], [17]. Following this approach, the present platform combines an Arduino Nano, a 12-bit MCP4725 digital-to-analog converter, an MCP602–IRLZ34N closed-loop current-regulation stage with a 10 Ω shunt resistor, and a 2N2222 active gate clamp. The system provides an experimentally evaluated current range of 0.50–40.00 mA. ON time, OFF time, cycle number, and current are configured locally through a 20 × 4 I2C LCD and three push-buttons, enabling standalone operation without an external computer. During the programmed relaxation interval, the active clamp establishes a hard-OFF condition to minimize residual current between pulses.

Pulsed Ag electrodeposition on anodized TiO_2_ was selected as an application-level validation because the resulting nanoparticle and dendritic morphologies are sensitive to current density, pulse timing, cycle number, and substrate geometry. The same hardware concept has previously been used to fabricate Ag nanoparticles and Ag dendrites on different TiO_2_ scaffolds for SERS applications [18], [19], [20]. In the present article, these cases are used to demonstrate operation under realistic low-voltage synthesis conditions; the resulting morphologies and analytical performance are examined in Section 7.3 rather than treated as the primary scope of the instrument.

The platform is not intended to replace commercial potentiostats or galvanostats for high-voltage, high-current, or potentiostatic studies. Instead, it is presented as a dedicated low-voltage tool for pulsed constant-current electrodeposition, particularly for protocols in which absolute current control, pulse timing, repeatable cycle execution, and a robust OFF state are sufficient. This article provides the circuit architecture, firmware, design files, bill of materials, assembly procedure, operation protocol, electrical characterization, and application-level validation required to reproduce and adapt the system.

## 2 Hardware description

### 2.1 Overall system architecture

The platform comprises five functional blocks: digital control, analog current command, current regulation, active gate clamping, and user interaction. An Arduino-compatible microcontroller manages the menu, pulse timing, state transitions, and safety actions. The MCP4725 digital-to-analog converter (DAC) converts the selected current into an analog voltage. This voltage is compared with the voltage across a 10 Ω shunt resistor by an MCP602 operational amplifier, which drives the gate of an IRLZ34N MOSFET. The MOSFET functions as the low-side current-control element. The electrochemical cell is operated in a two-electrode configuration, with the Pt anode connected to the 5 V line and the substrate cathode connected to the IRLZ34N drain; during electrical validation, the cell may be replaced by a test LED load. The MOSFET source connects to the shunt resistor, which returns to common ground. The shunt voltage is proportional to the load current and serves as the feedback signal. Once the feedback loop stabilizes, the current is determined by the DAC command and the shunt resistance. The IRLZ34N was selected due to its wide availability and compatibility as a logic-level N-channel MOSFET with the 5 V control range of this Arduino-based platform. In this circuit, the MOSFET is not used solely as a digital switch; rather, it acts as the pass element of the low-side analog current regulator, with its gate voltage continuously adjusted by the MCP602 operational amplifier. The TO-220 package facilitates prototyping and provides sufficient robustness for the evaluated low-current range. Under the present operating conditions, the MOSFET was evaluated up to 40.00 mA, resulting in low power dissipation. If the design is scaled to higher currents or higher cell voltages, MOSFET power dissipation, thermal management, and safe operating area must be reassessed.

This low-side topology was chosen due to its compatibility with inexpensive and widely available components. It also simplifies current sensing, as the shunt voltage is referenced to ground. The primary trade-off is that the electrochemical cell must operate within the limited voltage compliance of the 5 V supply, after accounting for voltage drops across the shunt resistor and MOSFET.

**Fig. 1:**
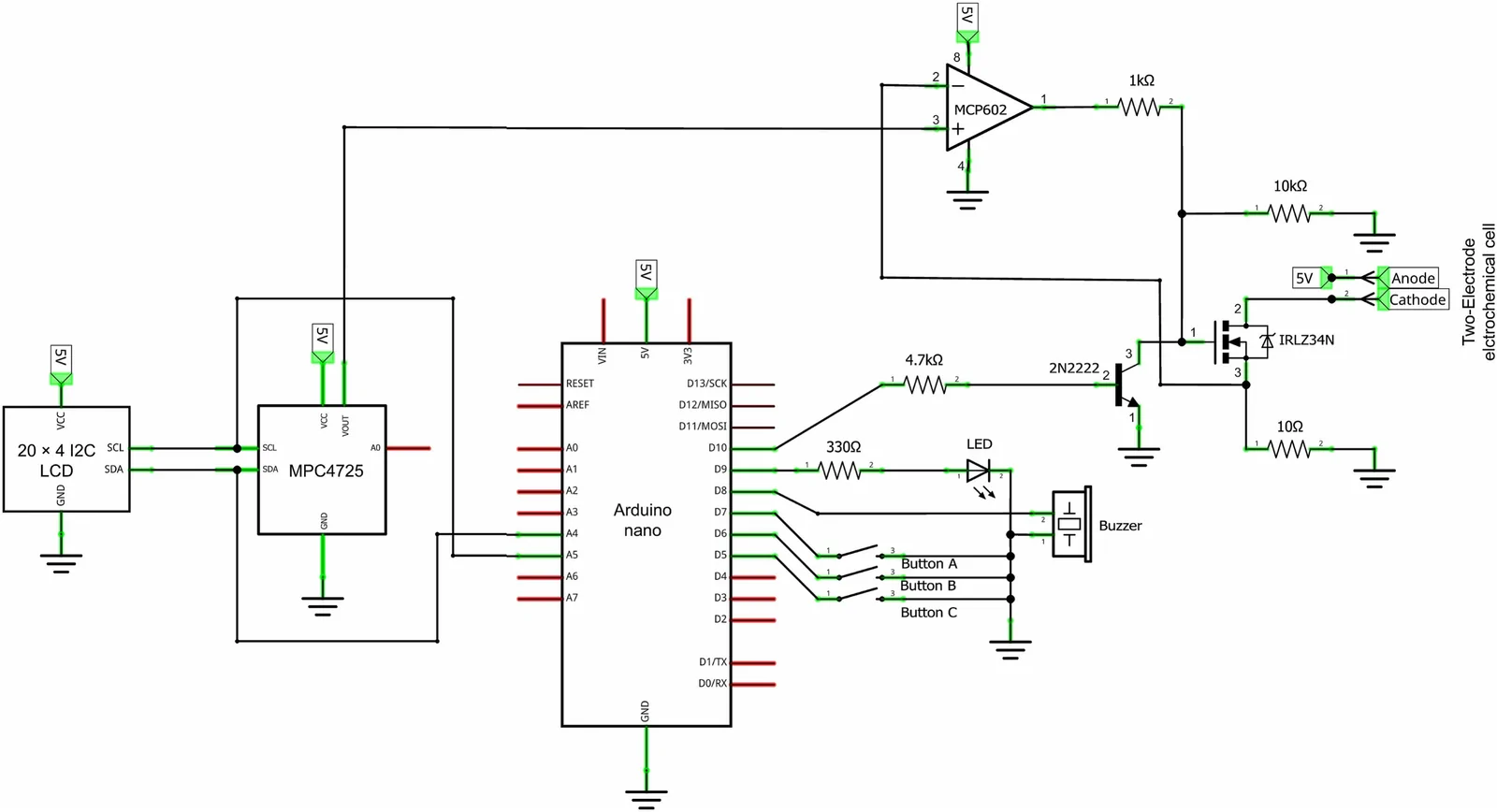
Complete electronic schematic of the Arduino-based pulsed constant-current platform.

Fig. 1 presents the complete electronic schematic of the platform. The diagram integrates the digital-control section, the current-regulation loop, the active gate-clamping stage, the user-interface components, and the external two-electrode electrochemical cell, while identifying the relevant pin assignments, component values, main current path, and feedback-sensing node.

### 2.2 Programmable reference and constant-current control loop

The analog loop regulates current by forcing the shunt voltage to match the DAC output (Eq (1):

(1) $$V_{\mathrm{DAC}}=V_{\mathrm{shunt}}$$

The output current is then estimated (Eq (2) as:

(2) $$I=\frac{V_{\mathrm{shunt}}}{R_{\mathrm{shunt}}}$$

where $R_{\mathrm{shunt}}$ is the current-sensing resistor. In this prototype, $R_{\mathrm{shunt}}$ = 10 Ω. Therefore, in the ideal case, currents of 0.50, 10.00, and 40.00 mA correspond to shunt voltages of 5.00, 100.00, and 400.00 mV, respectively. The DAC command voltage is calculated in the firmware (Eq (3) as:

(3) $$V_{\mathrm{DAC}}=I_{\mathrm{set}}R_{\mathrm{shunt}}$$

where $I_{\mathrm{set}}$ is the current selected by the user. The firmware measures the actual microcontroller supply voltage using the Arduino internal reference and adjusts the DAC output accordingly. This approach minimizes errors resulting from deviations of the logic supply from the nominal 5.00 V value. A 10 Ω shunt resistor was selected to balance current-sensing resolution and voltage compliance. Within the evaluated range of 0.50 to 40.00 mA, this resistor produces feedback voltages from 5.00 to 400.00 mV. These voltages are sufficient for resolution by the DAC–operational-amplifier feedback loop and low enough to preserve most of the 5.00 V supply for the electrochemical cell and MOSFET regulation. Lower shunt values, such as 1 Ω, would decrease voltage loss but generate sub-millivolt feedback signals at low currents, increasing susceptibility to offset and noise. In contrast, higher values, such as 50 to 100 Ω, would enhance current resolution but consume a significant portion of the available voltage at higher current setpoints. The 10 Ω shunt resistor is also compatible with the 12-bit MCP4725 DAC. With a 5.00 V reference, the DAC step size is approximately 1.22 mV, corresponding to an ideal current resolution of about 0.12 mA per DAC count. This resolution is finer than the 0.50 mA current step implemented in the firmware, and the shunt voltage remains limited to 400.00 mV at the maximum evaluated current of 40.00 mA. Therefore, the 10 Ω shunt resistor offers a practical compromise between current resolution and voltage compliance. Since the electrochemical cell is powered by the same 5.00 V supply, the voltage drop across the shunt and MOSFET must be sufficient to drive the electrochemical reaction and maintain feedback regulation. This configuration is suitable for low-voltage Ag electrodeposition experiments, which typically require 1 to 3 V under the conditions evaluated in this study.

### 2.3 Firmware, hard-OFF operation, and user interface

**Fig. 2:**
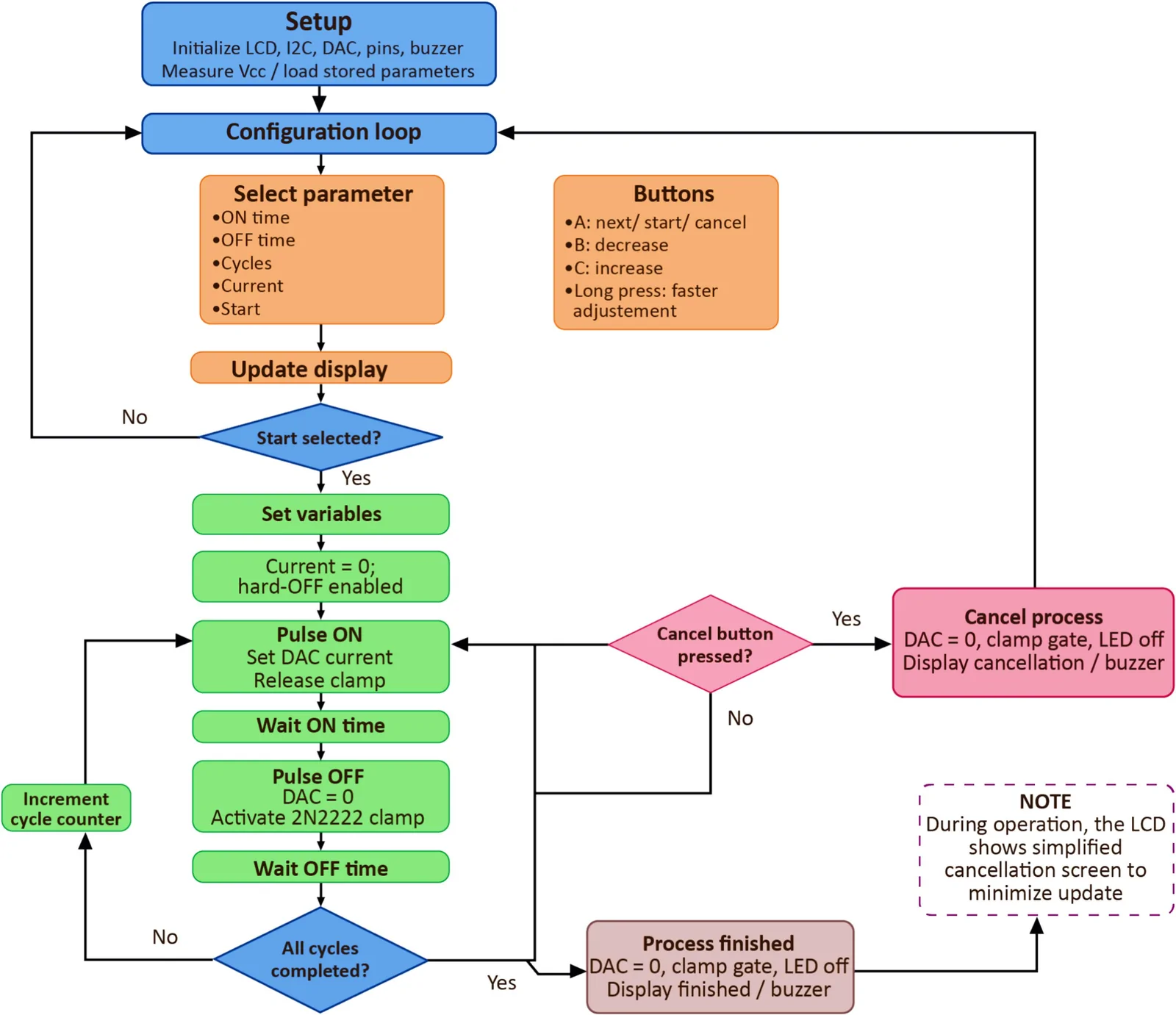
Flow diagram of the firmware implemented in the Arduino-based pulsed constant-current platform, showing the configuration loop, parameter selection, pulse execution, cycle counting, cancellation routine, and hard-OFF actions during the OFF interval.

The firmware operates as a finite-state machine comprising four states: configuration, processing, completion, and cancellation. During the configuration state, users select the ON time, OFF time, number of cycles, and current. In the processing state, the firmware executes the programmed pulse train with millisecond-scale timing control suitable for the evaluated electrodeposition protocols. Upon completion or cancellation, the system returns to the configuration menu, resetting the current setting to zero. The user interface comprises a 20 × 4 LCD connected through an I2C backpack, which provides the SDA and SCL interface shared with the MCP4725, together with three push-buttons. Button A is used to change the selected parameter and to initiate the process when the Start option is chosen. Buttons B and C decrease or increase the selected value, respectively. Long presses enable accelerated adjustment modes, facilitating both precise control and rapid navigation across extensive parameter ranges. During pulse execution, the LCD displays only the cancellation option to minimize unnecessary display updates. An LED connected to D9 indicates the ON phase of each pulse, while a buzzer connected to D8 provides acoustic feedback at the end of the process or following cancellation. The firmware workflow is illustrated in Fig. 2. Following initialization, the program enters a configuration loop where users select the ON time, OFF time, number of cycles, current setpoint, and start command via the three-button interface. Upon initiation, the firmware sets the necessary variables, establishes a safe zero-current condition, and executes the programmed pulse sequence by alternating between Pulse ON and Pulse OFF states. During Pulse ON, the DAC output is adjusted to the selected current and the gate clamp is disengaged. During Pulse OFF, the DAC command is set to zero and the 2N2222 clamp is activated to force the MOSFET gate into the OFF state. The cycle counter increments until the specified number of cycles is reached. At any stage during execution, the user may cancel the process, which returns the system to zero current before re-entering the configuration loop.

For firmware-level customization, the source file groups the editable settings under the section USER-CONFIGURABLE DEPOSITION SETTINGS. The variables onTime, offTime, ciclosMax, and corrienteCC define the values displayed at startup, whereas the constants ON_TIME_MIN_S, ON_TIME_MAX_S, OFF_TIME_MIN_S, OFF_TIME_MAX_S, CYCLES_MIN, CYCLES_MAX, CURRENT_MIN_ACTIVE_MA, and CURRENT_MAX_MA define the allowable ranges. The corresponding time, cycle, and current step-size constants may be edited to modify the adjustment increments applied by the push-buttons. After editing these values, the firmware must be recompiled and uploaded to the Arduino Nano. The hardware-calibration constant RSHUNT_OHM should only be changed to the measured resistance of the installed shunt; changes to the shunt resistor, supply voltage, maximum current, or power stage require revalidation of the current calibration, voltage compliance, and MOSFET power dissipation.

Beyond the TiO_2_/Ag SERS application used for validation in this work, the hardware can support other laboratory tasks within its validated low-voltage and low-current operating range:

• Pulsed electrodeposition and electrochemical surface modification: the platform can be adapted to deposit or modify conductive materials when controlled current pulses and relaxation intervals are required.
• Systematic process optimization: independently adjustable current, ON time, OFF time, and cycle number enable parameter screening for electrodeposition protocols without requiring a dedicated commercial workstation.
• Parallel and distributed experimentation: the low component cost and standalone operation facilitate the construction of multiple units for simultaneous synthesis experiments or comparative parameter studies.
• Teaching and prototyping: the open circuit, firmware, and breadboard architecture provide an accessible platform for demonstrating closed-loop current regulation, pulse generation, electrochemical control, and modification of laboratory hardware.

## 3 Design files summary

.

**Table:** 

**Design file name**	**File type**	**Open-source license**	**Location of the file**
Firmware_v1.1	INO	MIT License	https://doi.org/10.5281/zenodo.20750169
Current validation	csv	CC BY 4.0	https://doi.org/10.5281/zenodo.20750169
Software flow diagram	png	CC BY 4.0	https://doi.org/10.5281/zenodo.20750169
Complete electronic schematic	png	CC BY 4.0	https://doi.org/10.5281/zenodo.20750169
LCD enclosure cover	STL	CERN-OHL-P-2.0	https://doi.org/10.5281/zenodo.20750169
Breadboard enclosure cover	STL	CERN-OHL-P-2.0	https://doi.org/10.5281/zenodo.20750169
Enclosure base	STL	CERN-OHL-P-2.0	https://doi.org/10.5281/zenodo.20750169

The *Firmware_v1.1* file contains the Arduino source code used to control the user interface, current setpoint, ON/OFF pulse timing, cycle execution, and hard-OFF operation of the platform.

The *Current validation* file contains the programmed and multimeter-indicated current values used for the current-output validation presented in Section 7.1.

The *Software flow diagram* file contains the firmware workflow shown in Fig. 2, including parameter configuration, pulse execution, cycle counting, cancellation, and OFF-state control.

The *Complete electronic schematic* file contains the complete circuit diagram of the platform, including the digital-control, current-regulation, active gate-clamping, user-interface, and two-electrode electrochemical-cell connections.

The *LCD enclosure cover* file contains the STL model used to 3D print the protective cover for the 20 × 4 LCD module.

The *Breadboard enclosure cover* file contains the STL model for the main protective cover, including openings for the Arduino Nano power input, the LED and anode/cathode leads, and access to Buttons.

The *Enclosure base* file contains the STL model used to 3D print the supporting base for the LCD cover and breadboard enclosure components.

## 4 Bill of materials summary

.

**Table:** 

**Designator**	**Component**	**Number**	**Cost per unit −currency**	**Total cost − currency**	**Source of materials**	**Material type**
Arduino Nano	Arduino Nano	1	$ 8.00	$ 8.00	Amazon	Electronic
MCP4725	MCP4725 DAC module	1	$ 3.00	$ 3.00	Amazon	Electronic
MCP602	MCP602 operational amplifier	1	$ 0.67	$ 0.67	Amazon	Electronic
IRLZ34N	IRLZ34N N-channel MOSFET	1	$ 0.72	$ 0.72	Amazon	Electronic
2N2222	2N2222 NPN transistor	1	$ 0.04	$ 0.04	Amazon	Electronic
LCD	20 × 4 I2C LCD display	1	$ 8.99	$ 8.99	Amazon	Electronic
Button A	Red 12 × 12 mm Push-button	1	$ 0.28	$ 0.28	Amazon	Electronic
Button B	Black 12 × 12 mm Push-button	1	$ 0.28	$ 0.28	Amazon	Electronic
Button C	Black 12 × 12 mm Push-button	1	$ 0.28	$ 0.28	Amazon	Electronic
Buzzer	Active buzzer	1	$ 0.28	$ 0.28	Amazon	Electronic
LED	Indicator LED	1	$ 0.03	$ 0.03	Amazon	Electronic
10 Ω resistor	Current-sensing shunt resistor	1	$ 0.04	$ 0.04	Amazon	Electronic
1 kΩ resistor	Resistor between MCP602 output and IRLZ34N gate	1	$ 0.04	$ 0.04	Amazon	Electronic
10 kΩ resistor	Resistor between IRLZ34N gate and GND	1	$ 0.04	$ 0.04	Amazon	Electronic
4.7 kΩ resistor	Resistor between Arduino D10 and 2 N2222 base	1	$ 0.04	$ 0.04	Amazon	Electronic
330 Ω resistor	LED current-limiting resistor	1	$ 0.04	$ 0.04	Amazon	Electronic
Breadboard	830-point solderless prototyping breadboard	1	$ 3.00	$ 3.00	Amazon	Electronic
Mini Breadboard	170-point solderless prototyping breadboard	1	$ 1.00	$ 1.00	Amazon	Electronic
BW	22 AWG wire, color black	1	$ 0.50	$ 0.50	Amazon	Electronic
RW	22 AWG wire, color red	1	$ 0.50	$ 0.50	Amazon	Electronic
Enclosure	3D-printed protective enclosure (3 parts)	1	$ 1.50	$ 1.50	Locally fabricated	Mechanical

## 5 Build instructions

### 5.1 Breadboard reference system and wiring convention

**Fig. 3:**
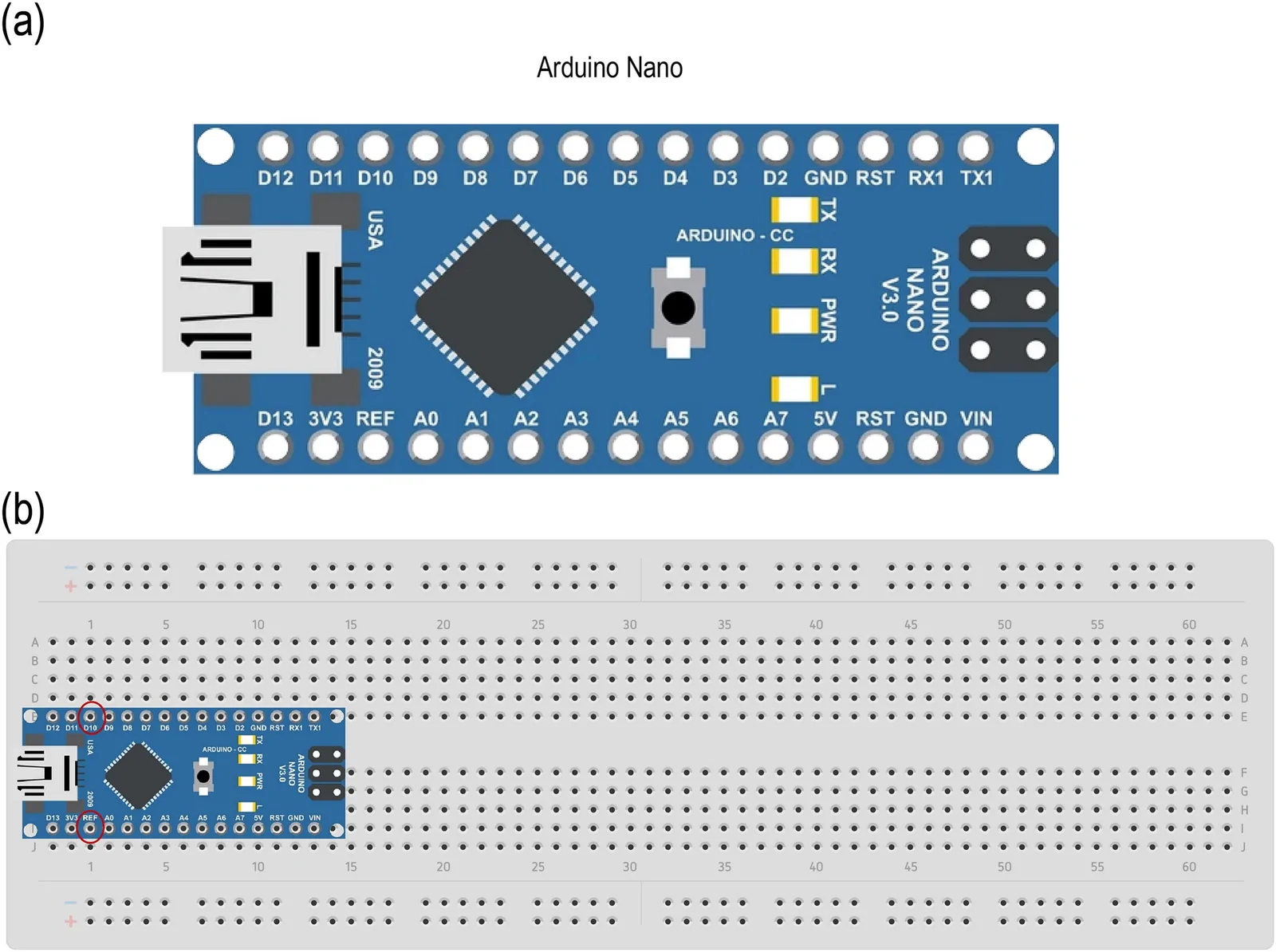
Breadboard coordinate reference used for the physical assembly of the Arduino-based pulsed constant-current platform. (a) Arduino pin reference showing D10 and REF. (b) Arduino placement on the 830-point breadboard, with Arduino pin D10 aligned with A1 and REF aligned with I1 (red circles). (For interpretation of the references to colour in this figure legend, the reader is referred to the web version of this article.)

The prototype was assembled on a standard 830-point solderless breadboard and an auxiliary 170-point mini breadboard for the 20 × 4 I2C LCD module. To ensure reproducibility of the physical layout, all connections are described using breadboard coordinates. The general coordinate reference used for the complete assembly is shown in Fig. 3. Fig. 3(a) shows the Arduino pin reference used to identify D10 and REF, whereas Fig. 3(b) shows the Arduino placement on the 830-point breadboard. The Arduino was used as the spatial reference for the assembly. In the final layout, Arduino pin D10 must be aligned with coordinate A1, and the reference point labeled REF must be aligned with coordinate I1. All subsequent component placements and jumper-wire connections are defined relative to this orientation. In the following instructions, RW denotes a red jumper wire used for positive-supply or signal-routing connections, whereas BW denotes a black jumper wire used for ground connections. The notation “Xn > Ym” indicates a jumper wire connecting coordinate Xn to coordinate Ym. The polarity and orientation of polarized components, including the LED, buzzer, MCP4725 module, LCD module, 2N2222 transistor, IRLZ34N MOSFET, and MCP602 operational amplifier, must be verified before powering the circuit.

### 5.2 Power-rail preparation

**Fig. 4:**
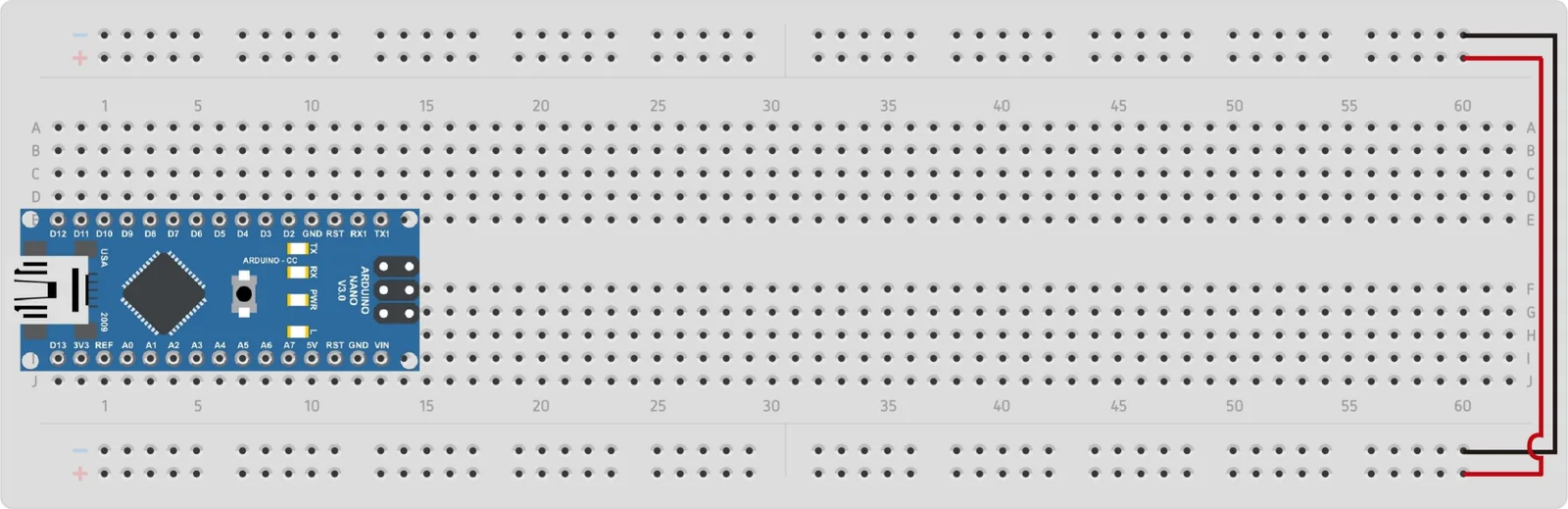
Power-rail preparation of the breadboard. The positive and ground rails were bridged from one end of the board to the other before installing the electronic components.

Before inserting or wiring the electronic components, the power rails of the breadboard were bridged from one end to the other to ensure continuous positive and ground distribution along the complete length of the prototyping board. The power-rail configuration is illustrated in Fig. 4, and the corresponding rail connections are summarized in Table 1.

**Table 1:** General breadboard rail connections.

Step	Connection	Description
1	RW (+60) – RW (+60)	Bridge the positive rail from one end of the breadboard to the other
2	BW (−60) – BW (−60)	Bridge the ground rail from one end of the breadboard to the other

After completing the rail connections listed before, continuity along both the positive and ground rails was verified using a multimeter. The absence of a short circuit between the positive and ground rails was also confirmed before installing the remaining components.

### 5.3 Arduino placement and wiring

**Fig. 5:**
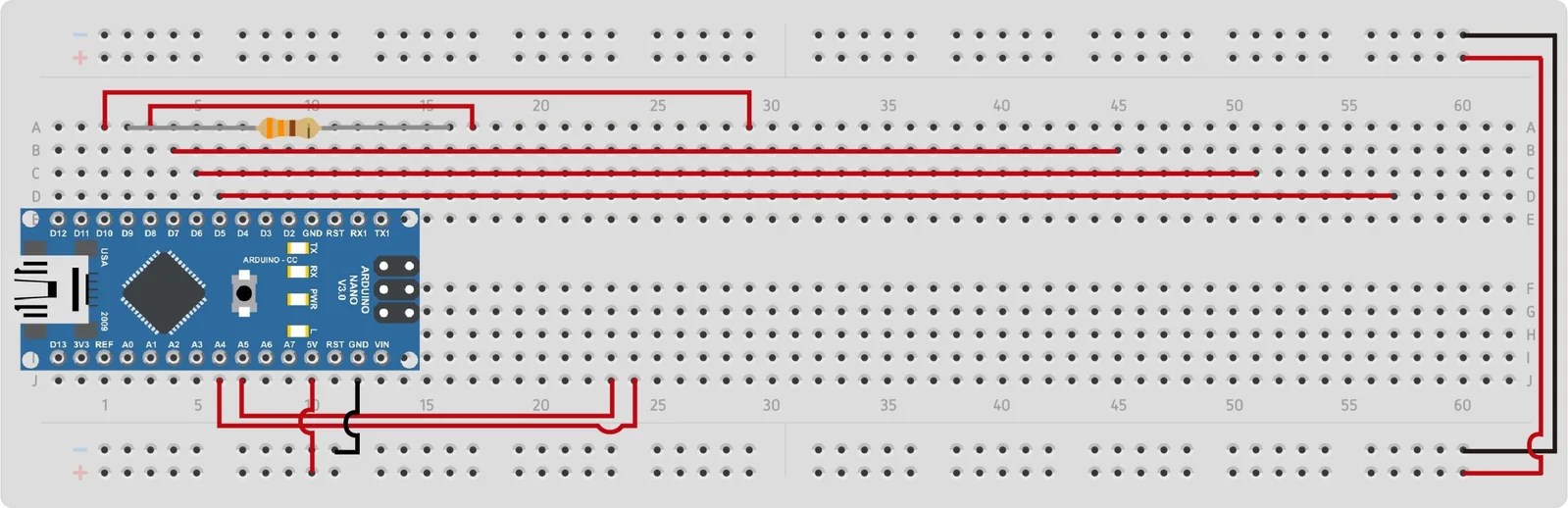
Arduino placement and wiring on the 830-point breadboard.

Place the Arduino on the breadboard according to the reference defined in Fig. 3. The board must be inserted so that the digital and analog pins align with the coordinates listed in Table 2. The Arduino placement and direct jumper-wire connections are shown in Fig. 5. After placement, connect the Arduino pins to the corresponding breadboard rows and power rails according to Table 2.

**Table 2:** Arduino breadboard-level connections.

Arduino pin	Arduino coordinate	Connection	Destination
D5	E6	RW	D6 > D57
D6	E5	RW	C5 > C51
D7	E4	RW	B4 > B45
D8	E3	RW	A3 > A17
D9	E2	330 Ω resistor	A2 > A16
D10	E1	RW	A1 > A29
A4	I6	RW	J6 > J24
A5	I7	RW	J7 > J23
5 V	I10	RW	J10 > +10
GND	I12	BW	J12 > -11

### 5.4 Push-button connections

**Fig. 6:**
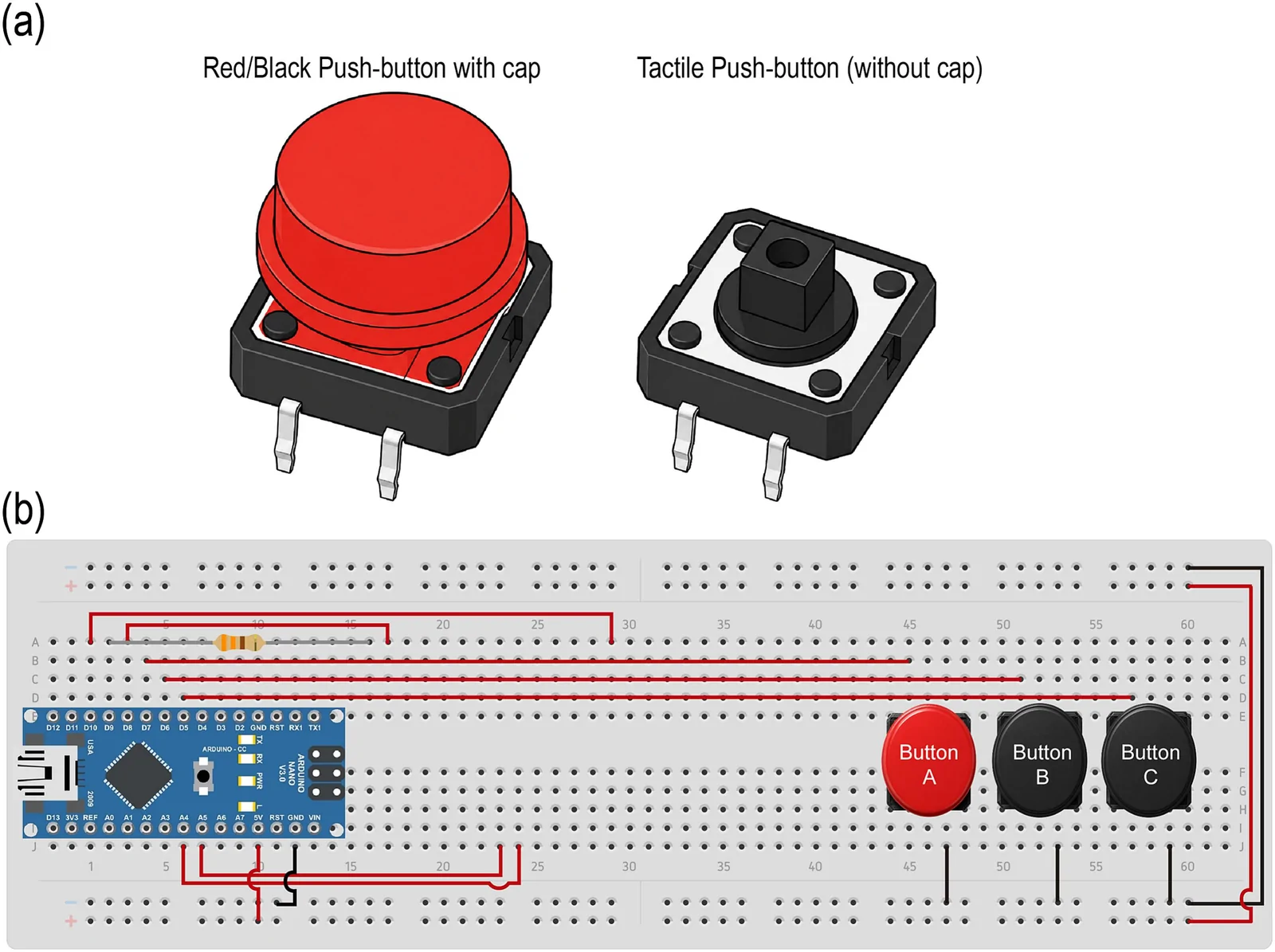
Push-button placement and wiring. (a) Push-button component with/without cap. (b) Breadboard placement and wiring of Button A, Button B, and Button C.

Install the three push-buttons on the right side of the main breadboard, as shown in Fig. 6. Fig. 6(a) shows the push-button component with and without cap, and Fig. 6(b) shows the corresponding breadboard wiring. Connect each button to the corresponding Arduino signal line and ground rail using the coordinates listed in Table 3.

**Table 3:** Push-button breadboard-level connections.

Component	Breadboard coordinate	Connection	Destination
Button A	E45	Button A opposite side	H47
Button A	H47	BW	J47 > -47
Button B	E51	Button B opposite side	H53
Button B	H53	BW	J53 > -53
Button C	E57	Button C opposite side	H59
Button C	H59	BW	J59 > -59

After wiring the push-buttons according to Fig. 6(b) and Table 3, verify that each button is normally open and that electrical continuity is established only when the corresponding button is pressed.

### 5.5 Buzzer and LED indicator connections

**Fig. 7:**
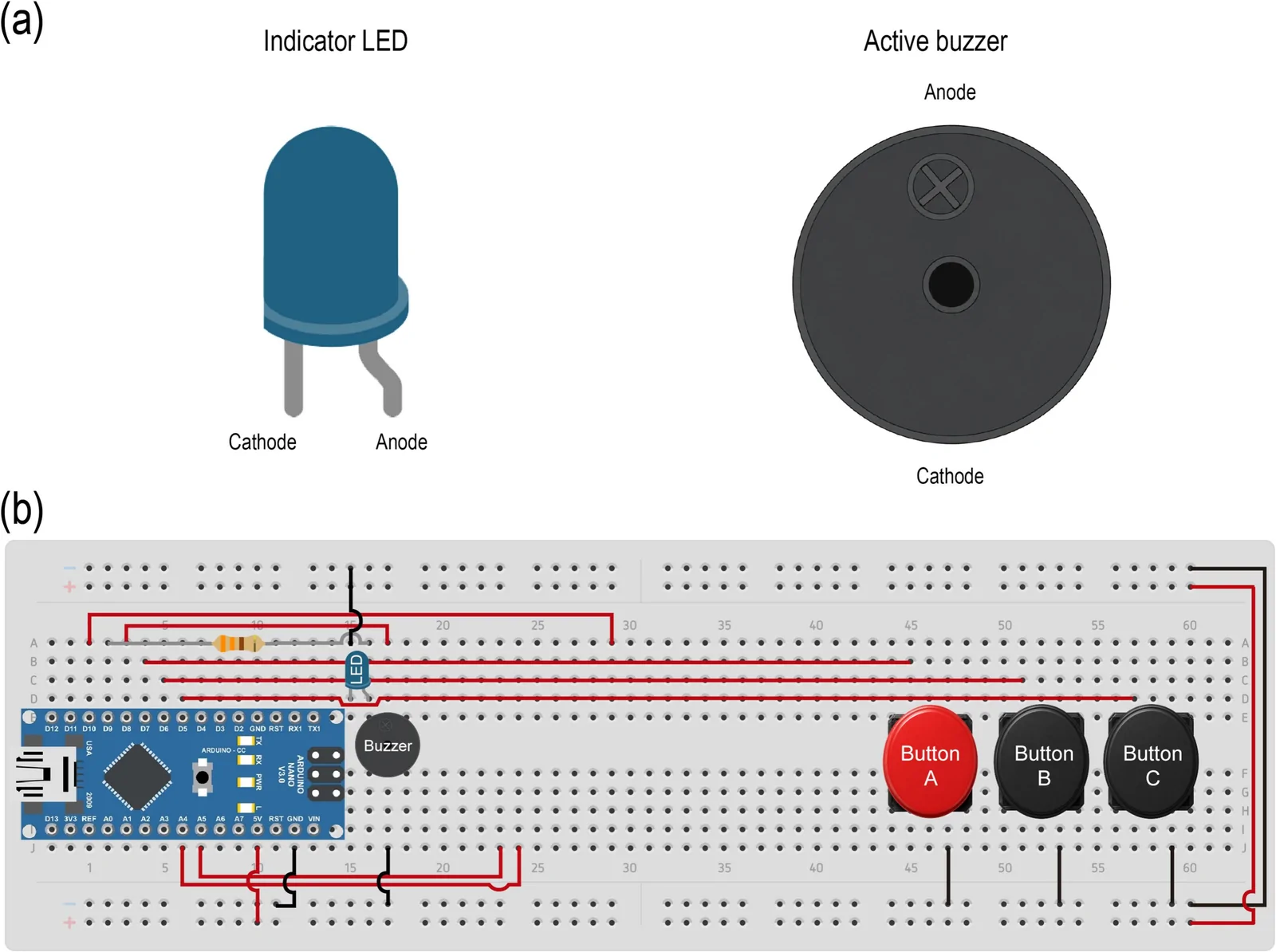
Buzzer and LED indicator wiring. (a) LED and buzzer polarity reference. (b) Breadboard wiring of the buzzer and LED indicator.

Install the buzzer and LED indicator near the Arduino, following the layout shown in Fig. 7. Fig. 7(a) shows the LED and buzzer components with their polarity reference, whereas Fig. 7(b) shows their placement and wiring on the breadboard. The buzzer must be connected with the correct polarity. The LED must be connected in series with the 330 Ω current-limiting resistor placed between A2 and A16, as specified in Table 2. The detailed buzzer and LED connections are listed in Table 4.

**Table 4:** Buzzer and LED breadboard-level connections.

Component	Breadboard coordinate	Connection	Destination
Buzzer anode	E17	Buzzer cathode	F17
Buzzer cathode	F17	BW	J17 > -29
LED anode	D16	LED cathode	D15
LED cathode	D15	BW	A15 > -15

Before powering the circuit, verify the LED and buzzer polarity shown in Fig. 7(a) and the wiring shown in Fig. 7(b). The LED anode must be connected toward the Arduino D9 signal path through the 330 Ω resistor, and the cathode must return to ground.

### 5.6 MCP4725 DAC module connections

**Fig. 8:**
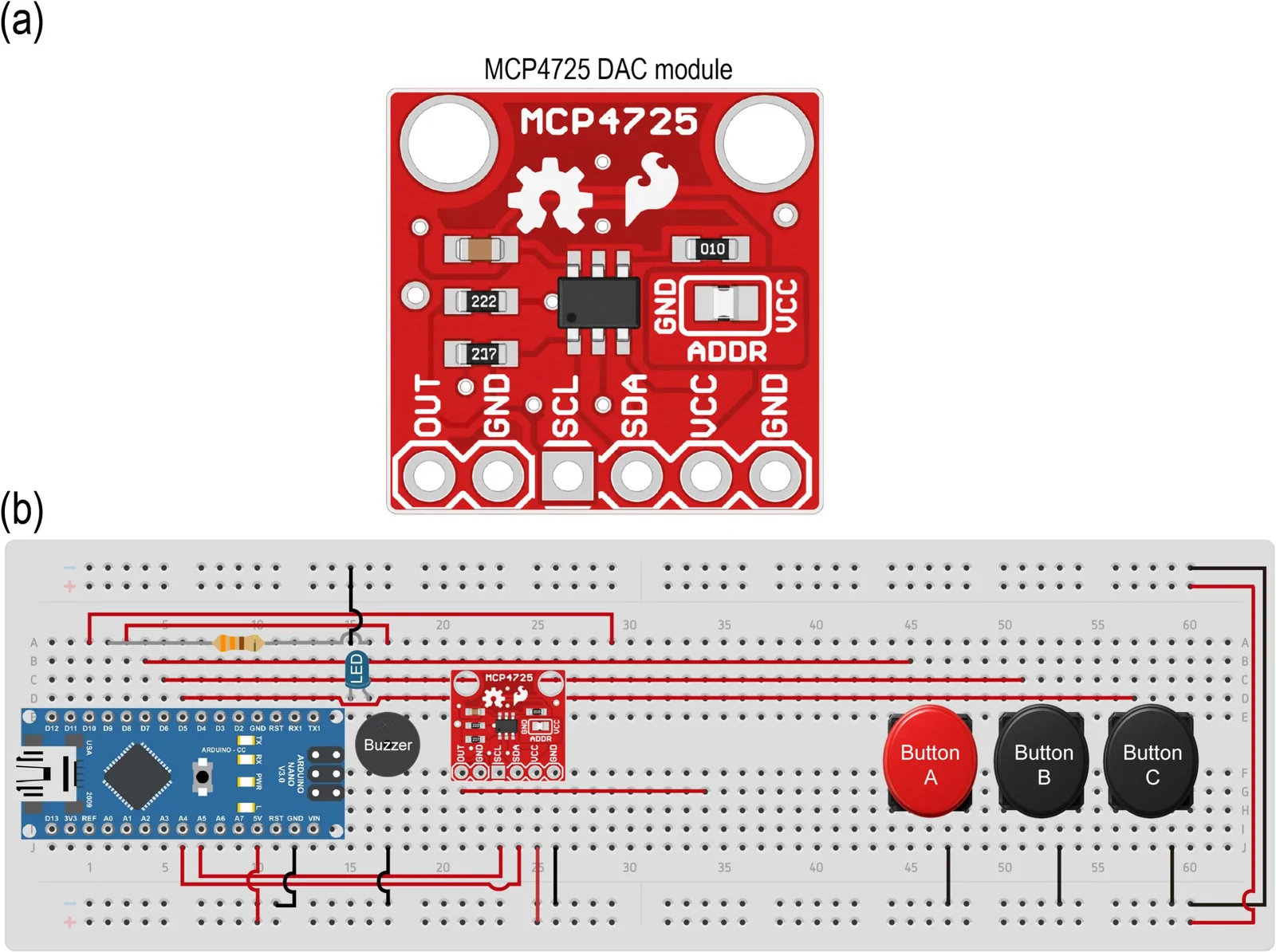
MCP4725 digital-to-analog converter wiring. (a) MCP4725 module pinout. (b) Breadboard placement and wiring of the MCP4725 module.

Place the MCP4725 module on the main breadboard with its pins aligned to the coordinates shown in Fig. 8. Fig. 8(a) shows the MCP4725 module pinout, and Fig. 8(b) shows the corresponding breadboard wiring. The MCP4725 connections are summarized in Table 5. The OUT pin is routed toward the analog control section.

**Table 5:** MCP4725 breadboard-level connections.

MCP4725 pin	Breadboard coordinate	Connection	Destination
OUT	F21	RW	G1 > G34
SCL	F23	I2C bus	Routed to Arduino A5
SDA	F24	I2C bus	Routed to Arduino A4
VCC	F25	RW	J25 > +25
GND	F26	BW	J26 > -26

### 5.7 2N2222 transistor connections

**Fig. 9:**
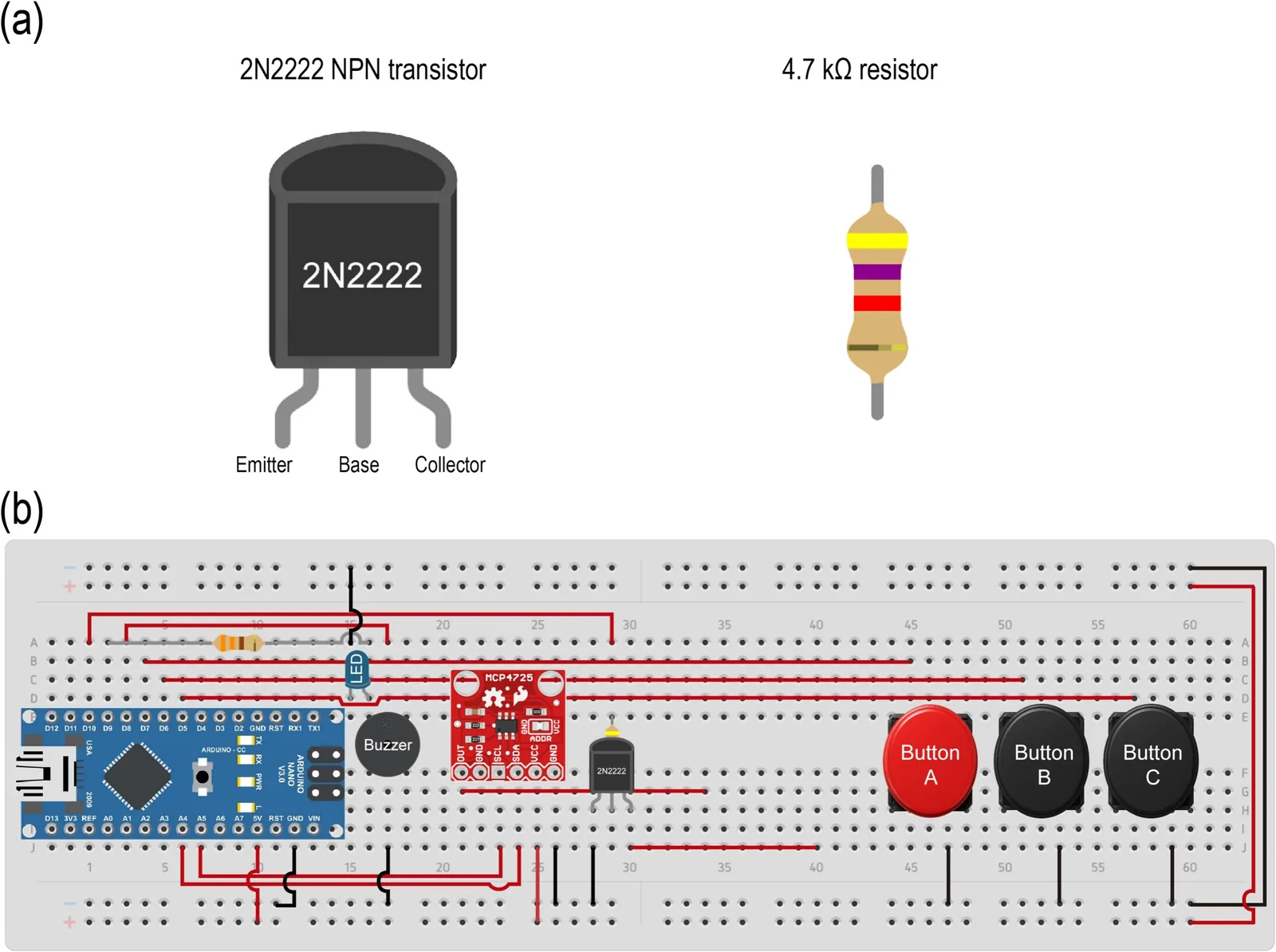
2N2222 transistor wiring. (a) 2N2222 transistor and 4.7 kΩ resistor reference. (b) Breadboard placement and wiring of the 2N2222 transistor.

Insert the 2N2222 transistor according to its package pinout and the placement shown in Fig. 9. Fig. 9(a) shows the 2N2222 transistor and the 4.7 kΩ resistor used in this section, whereas Fig. 9(b) shows their breadboard placement and wiring. The emitter, base, and collector must match the coordinates listed in Table 6. Because 2N2222 pin ordering can vary depending on manufacturer and package, verify the pinout from the specific component datasheet before insertion.

**Table 6:** 2N2222 breadboard-level connections.

2N2222 terminal	Breadboard coordinate	Connection	Destination
Emitter	H28	BW	J28 > -28
Base	H29	4.7 kΩ resistor	F29 > E29
Collector	H30	RW	J30 > J40

After installing the transistor according to Fig. 9(b) and Table 6, verify that the 4.7 kΩ resistor is connected between the Arduino D10 signal path and the 2N2222 base.

### 5.8 IRLZ34N breadboard-level connections

**Fig. 10:**
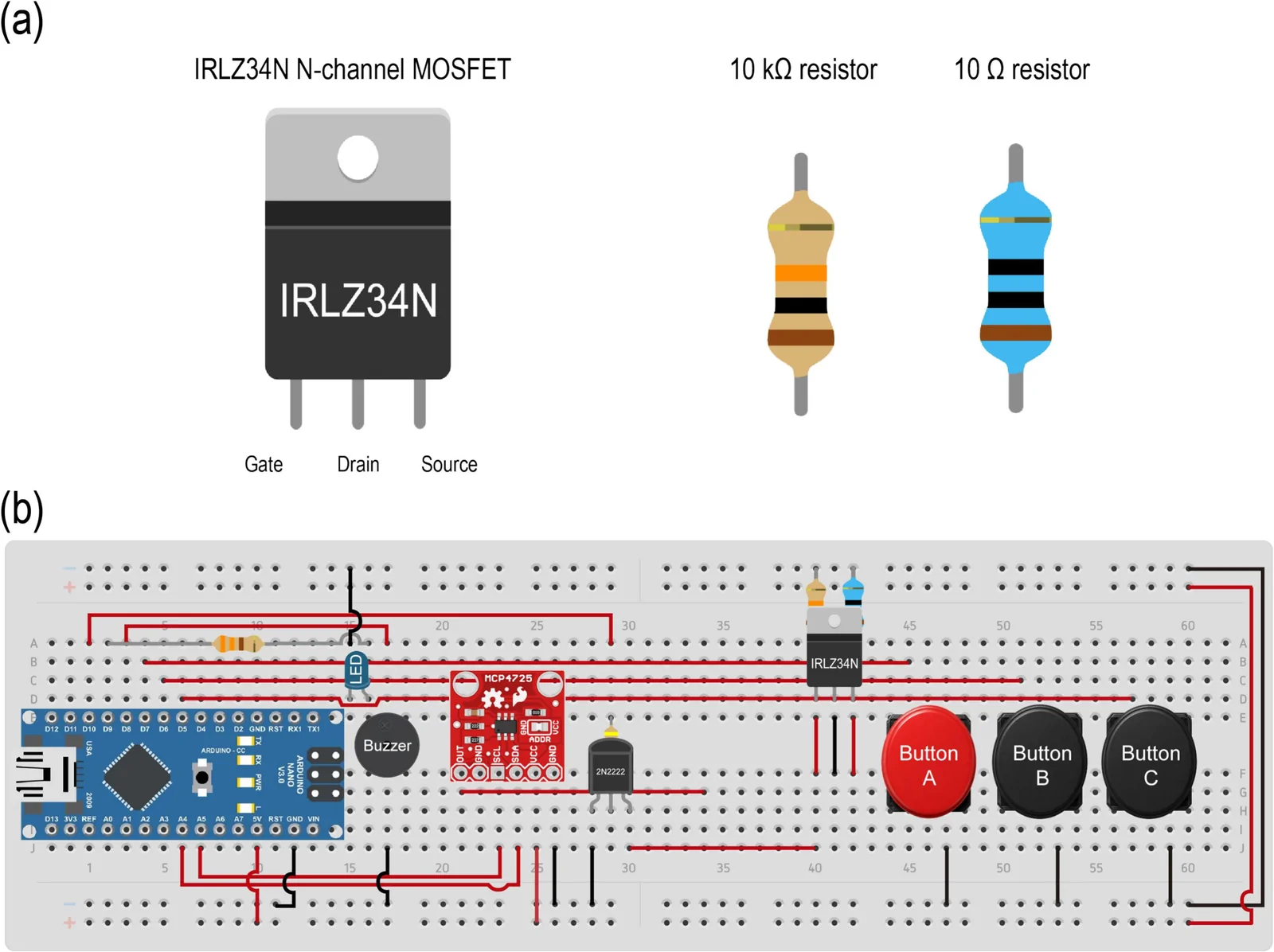
IRLZ34N MOSFET and shunt-resistor wiring. (a) IRLZ34N, 10 kΩ resistor, and 10 Ω shunt-resistor reference. (b) Breadboard placement and wiring of the IRLZ34N, 10 Ω shunt resistor, and MOSFET gate pull-down resistor.

Insert the IRLZ34N using the TO-220 pinout specified by the manufacturer. For the IRLZ34N package used in this prototype, the pins must be assigned as gate, drain, and source according to the layout shown in Fig. 10 and the coordinates listed in Table 7. Fig. 10(a) shows the IRLZ34N and the associated resistors used in this section, whereas Fig. 10(b) shows their breadboard placement and wiring. Verify the IRLZ34N orientation before powering the circuit.

**Table 7:** IRLZ34N breadboard-level connections.

IRLZ34N terminal	Breadboard coordinate	Connection	Destination
Gate	D40	10 kΩ resistor	A40 > -40
Gate	D40	RW	E40 > F40
Drain	D41	Cathode/low-side node jumper	E41 > F41
Source	D42	10 Ω resistor	A42 > -42
Source	D42	RW	E42 > F42

he MOSFET drain node shown in Fig. 10(b) is the connection point for the cathode side of the external test load or electrochemical cell. The positive side of the load or electrochemical cell must be connected to the positive supply rail. The source node must be connected to the 10 Ω shunt resistor, and the opposite side of the shunt resistor must return to ground, as indicated in Table 7.

### 5.9 MCP602 operational-amplifier connections

**Fig. 11:**
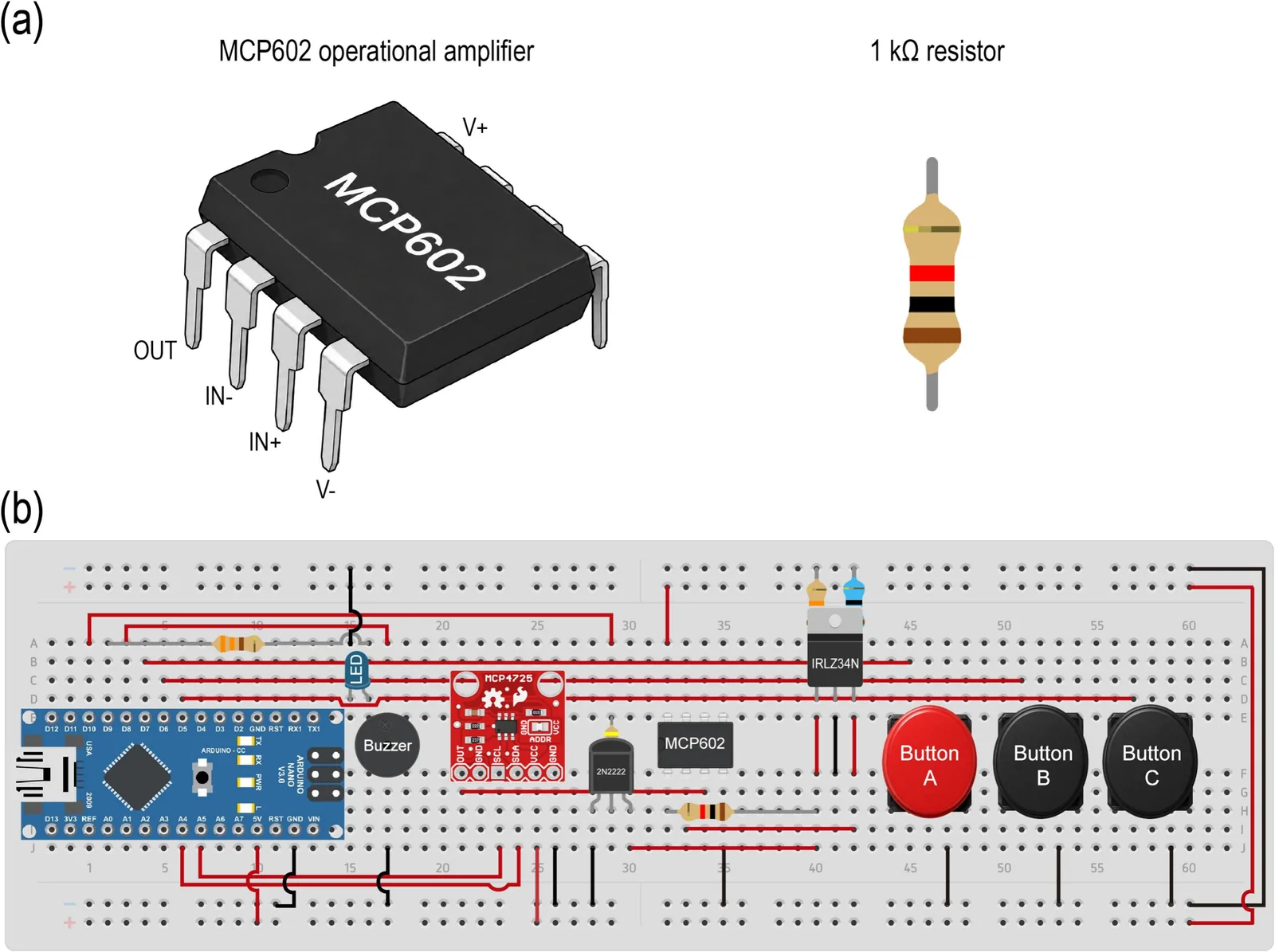
MCP602 operational-amplifier wiring. (a) MCP602 operational amplifier and associated resistor reference. (b) Breadboard placement and wiring of the MCP602 operational amplifier.

Insert the MCP602 operational amplifier across the central gap of the breadboard, ensuring correct pin-1 orientation. The notch or dot on the integrated circuit package must be used to identify pin 1 before insertion. The MCP602 placement is shown in Fig. 11, and its breadboard-level connections are listed in Table 8. Fig. 11(a) shows the MCP602 and the associated resistor used in this section, whereas Fig. 11(b) shows the MCP602 placement and wiring on the breadboard.

**Table 8:** MCP602 breadboard-level connections.

MCP602 pin	Breadboard coordinate	Connection	Destination
OUT	F32	1 kΩ resistor	H32 > H40
IN−	F33	RW	I33 < I42
IN+	F34	Signal node	MCP4725 OUT line
V−	F35	BW	J35 < -35
V+	E32	RW	A32 < +32

After wiring the MCP602 according to Fig. 11(b) and Table 8, verify that the IN + input is connected to the MCP4725 OUT node listed in Table 5, and that the IN − input is routed to the current-sensing shunt node listed in Table 7.

### 5.10 LCD module on auxiliary mini breadboard

**Fig. 12:**
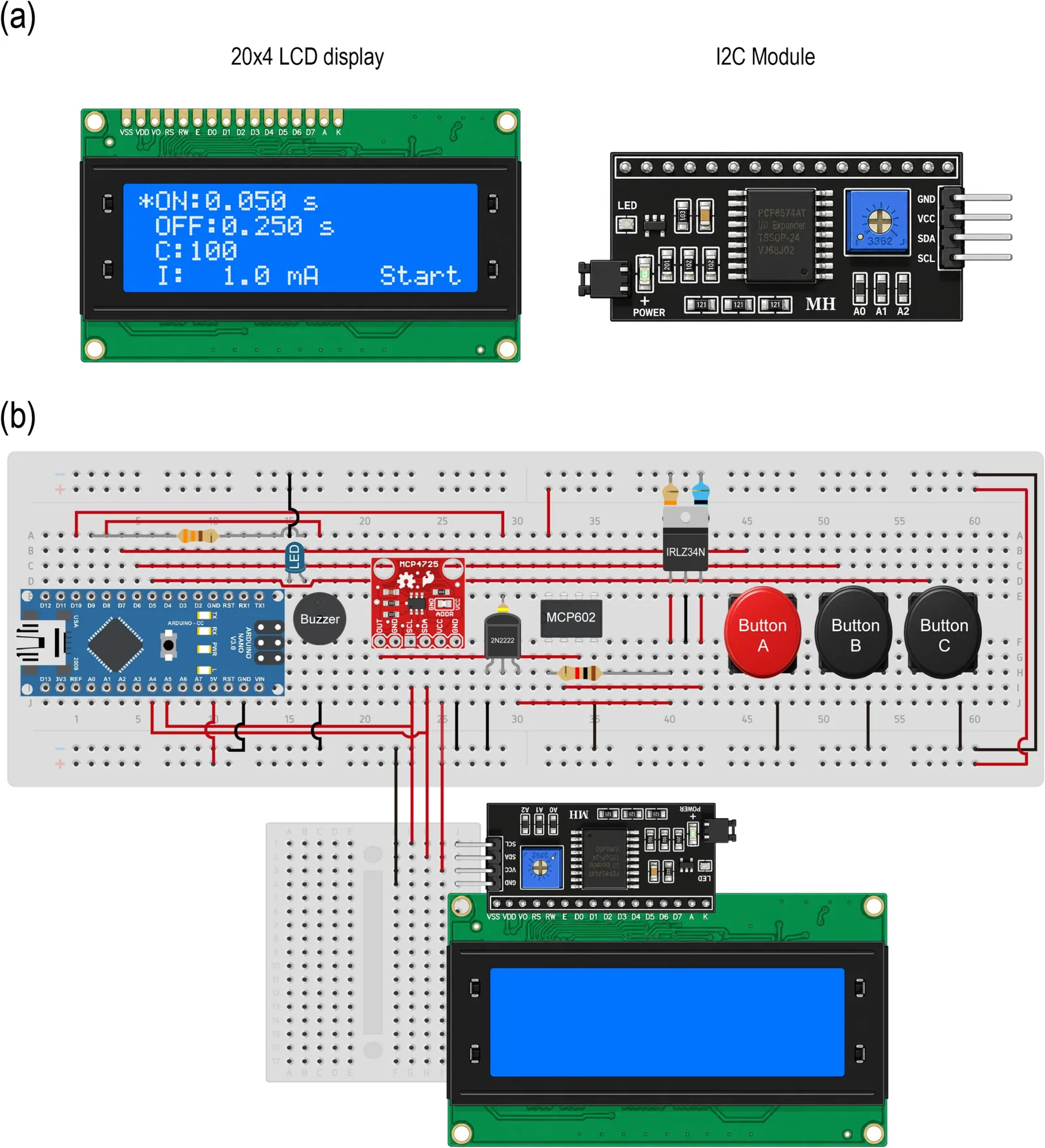
LCD module wiring. (a) 20 × 4 LCD module and I2C backpack pin reference. (b) Wiring of the LCD module from the auxiliary mini breadboard to the main 830-point breadboard.

Mount the 20 × 4 I2C LCD module on the auxiliary 170-point mini breadboard, as shown in Fig. 12. Fig. 12(a) shows the LCD module and I2C backpack pin reference, whereas Fig. 12(b) shows the LCD wiring from the auxiliary mini breadboard to the main 830-point breadboard. The LCD module was wired to the main 830-point breadboard through four jumper wires corresponding to SCL, SDA, VCC, and GND. The corresponding connections are listed in Table 9. In this table, the “Mini breadboard coordinate” column indicates the physical pin position of the LCD module on the auxiliary mini breadboard, whereas the “Destination” column indicates the jumper route from the auxiliary mini breadboard to the main 830-point breadboard.

**Table 9:** LCD module connections between the auxiliary mini breadboard and the main 830-point breadboard.

LCD pin	Mini breadboard coordinate	Connection	Destination (mini breadboard > breadboard)
SCL	J1	RW	G1 > I23
SDA	J2	RW	H2 > I24
VCC	J3	RW	I3 > +25
GND	J4	BW	F4 > –22

After wiring the LCD according to Fig. 12(b) and Table 9, verify that the LCD VCC and GND lines are connected to the same power and ground rails used by the Arduino and MCP4725 module. Also verify that the SCL and SDA lines are continuous between the auxiliary mini breadboard and the I2C bus on the main 830-point breadboard.

### 5.11 Final wiring inspection before powering

The complete assembled prototype is shown in Fig. 13. Before connecting the Arduino to USB power, inspect the complete assembly and compare it with the individual subsection layouts shown in Fig. 3, Fig. 4, Fig. 5, Fig. 6, Fig. 7, Fig. 8, Fig. 9, Fig. 10, Fig. 11, Fig. 12 and the connection lists provided in Table 1, Table 2, Table 3, Table 4, Table 5, Table 6, Table 7, Table 8, Table 9.

**Fig. 13:**
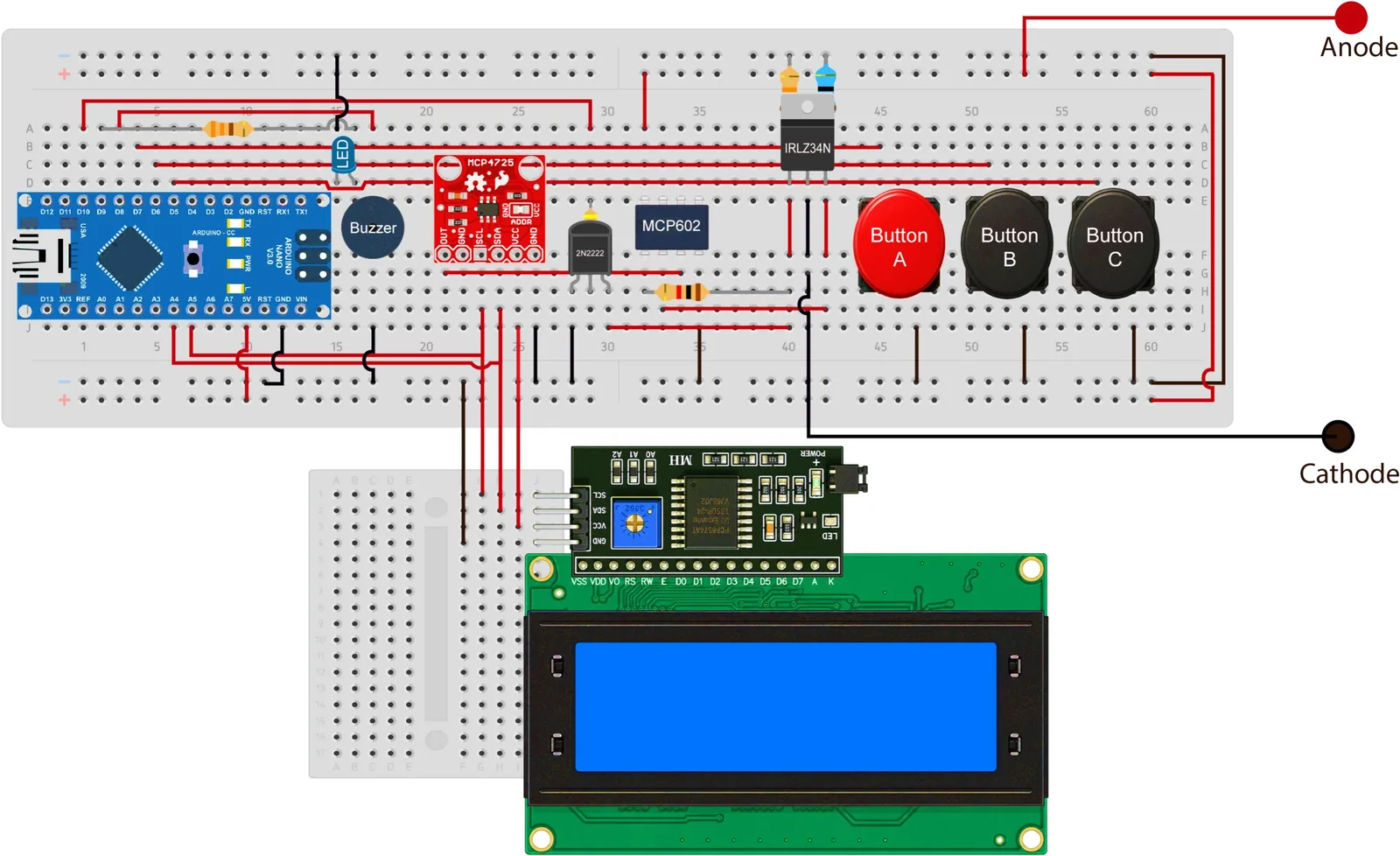
Complete breadboard-level assembly of the Arduino-based pulsed constant-current platform before connection to a test load or electrochemical cell.

Before the first test, two external connection leads must be installed. The positive lead must be connected from any available point of the positive supply rail (+) to the anode side of the external load. This external load may be a dummy load, a test LED, or the electrochemical cell. The negative lead must be connected from the IRLZ34N drain node to the cathode side of the same external load. Therefore, during operation, the external load or electrochemical cell is connected between the positive supply rail and the IRLZ34N drain node.

The final inspection was performed as follows:

• Verify that the positive and ground rails shown in Fig. 4 are not shorted.
• Verify continuity along the positive and ground rails listed in Table 1.
• Confirm that all ground-referenced components return to the common ground rail.
• Confirm the polarity of the buzzer and LED shown in Fig. 7(a) and the wiring shown in Fig. 7(b).
• Confirm the orientation and wiring of the three push-buttons shown in Fig. 6(a) and Fig. 6(b).
• Confirm the pin orientation of the 2N2222 transistor shown in Fig. 9(a) and the wiring shown in Fig. 9(b).
• Confirm the gate, drain, and source orientation of the IRLZ34N MOSFET shown in Fig. 10(a) and the wiring shown in Fig. 10(b).
• Confirm the pin-1 orientation of the MCP602 operational amplifier shown in Fig. 11(a) and the wiring shown in Fig. 11(b).
• Confirm that the MCP4725 and LCD share the same SDA and SCL lines, as listed in Table 5, Table 9 and shown in Fig. 8(b) and 12(b).
• Confirm that the external anode lead is connected from an available positive-rail point (+) to the anode side of the dummy load, test LED, or electrochemical cell.
• Confirm that the external cathode lead is connected from the IRLZ34N drain node shown in Fig. 10(b) to the cathode side of the dummy load, test LED, or electrochemical cell.
• Confirm that the dummy load, test LED, or electrochemical cell is connected only between the positive supply rail and the IRLZ34N drain node.
• Confirm that no liquid electrochemical cell is connected during the first electrical test.

Only after completing the inspection procedure described above should the Arduino be connected to USB power. The first test must be performed with a dummy load or test LED, not with the electrochemical cell.

### 5.12 3D-printed protective enclosure

**Fig. 14:**
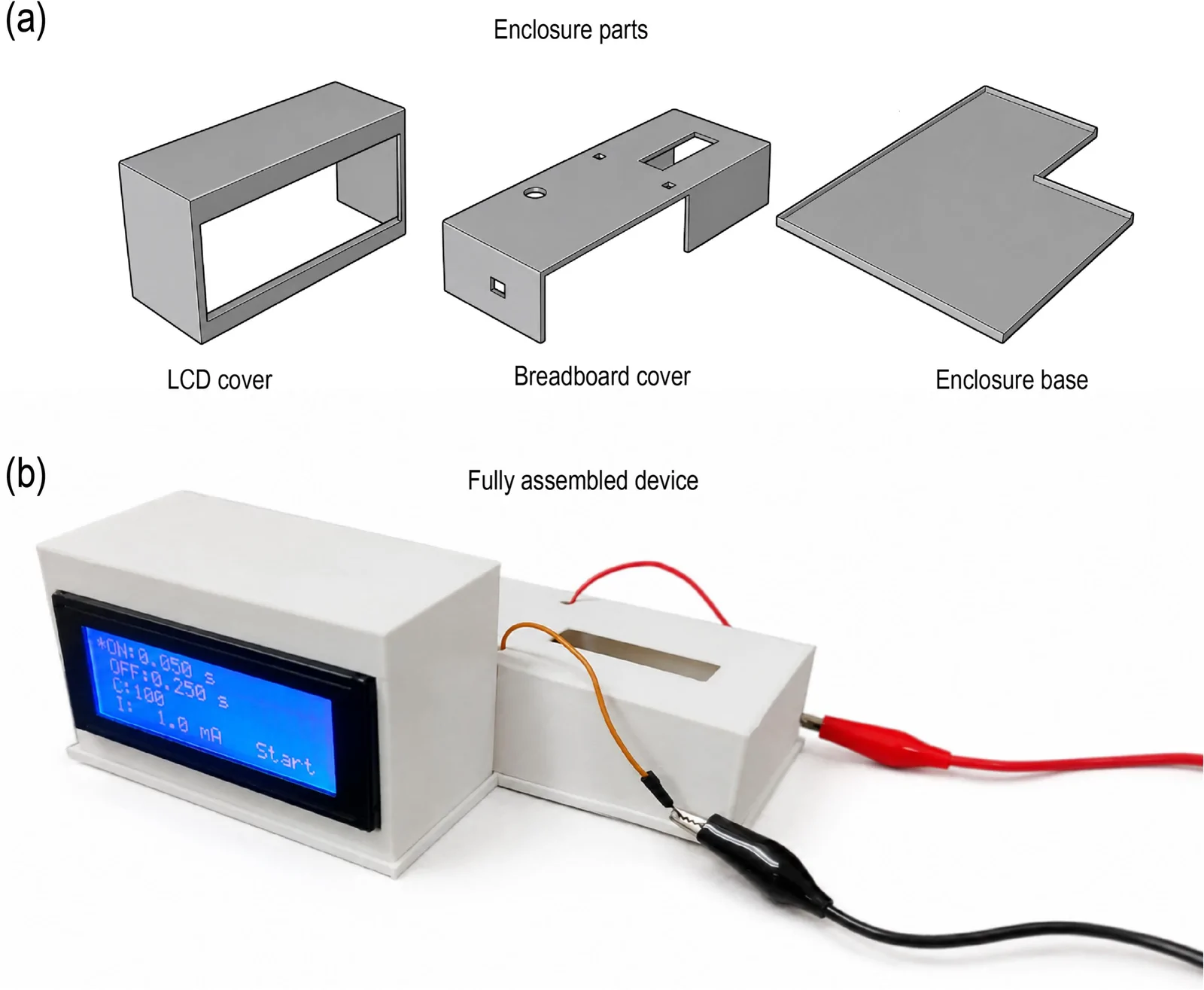
3D-printed protective enclosure for the pulsed constant-current platform. (a) Individual enclosure components: LCD cover, breadboard cover, and supporting base. (b) Fully assembled device with the protective enclosure installed.

A 3D-printed protective enclosure was developed to reduce the exposure of the breadboard electronics during operation in a wet-chemical laboratory environment. The enclosure consists of three parts: (i) a dedicated cover for the LCD module, (ii) a main cover for the breadboard assembly, and (iii) a base supporting both upper parts. The breadboard cover includes an opening for the Arduino Nano power-input connection, three outlet holes for the led and external wires connected to the anode and cathode, and a opening that allows access to Buttons A–C. The enclosure parts can be printed in PETG or PLA using a 0.4 mm nozzle, a printing temperature of 230 °C, and a heated bed at 80 °C. The total printing time is approximately 2.5 h and the total material consumption is about 50 g. After printing, the LCD cover and the breadboard cover are positioned on the base to protect the electronics while maintaining access to the display, buttons, power input, and electrode leads. If a permanent assembly is desired, the parts may be fixed in place using silicone adhesive. The fully assembled device with the protective enclosure is shown in Fig. 14.

## 6 Operation instructions

### 6.1 Pre-operation checklist

Before each experiment, ensure the cell is compatible with the available voltage compliance. Confirm the current setpoint is between 0.50 and 40.00 mA, the shunt resistor value matches the firmware constant, and the electrolyte does not contact the electronics. Verify the electrochemical cell polarity: connect the Pt anode to 5 V and the substrate cathode to the MOSFET drain.

### 6.2 Parameter configuration

After startup, the LCD displays the configurable parameters. Use Button A to navigate through ON time, OFF time, cycles, current, and Start. Use Buttons B and C to decrease or increase the selected value. Holding B or C enables faster adjustments. Table 10 summarizes a representative Ag electrodeposition program for fabricating a TiO_2_/Ag SERS substrate.

**Table 10:** Representative pulsed-current electrodeposition parameters for Ag deposition on anodized TiO_2_ substrates.

Parameter	Value
ON time	50 ms
OFF time	250 ms
Cycles	400
Target current density	5 mA/cm^2^
Exposed cathode area	User-defined
Programmed current	I = J x A
Electrolyte	10 mM AgNO_3_ + 100 mM NaNO_3_
Anode	Pt sheet
Cathode	Anodized TiO_2_ substrate

The firmware parameter labeled as current refers to the absolute current delivered by the platform in mA, not current density. If your electrodeposition protocol specifies mA/cm^2^, calculate the required current using I = J × A, where J is the target current density and A is the exposed electrode area. Enter the calculated current value into the device.

### 6.3 Running and canceling a process

To begin deposition, select Start and press Button A. The LED illuminates during each ON pulse and turns off during the OFF interval. The LCD shows the cancellation screen to prevent unnecessary updates during pulse generation. To cancel, press Button A while the process is running. The firmware will set the DAC output to zero, activate the 2N2222 gate clamp, turn off the LED, sound the buzzer, and return to the configuration menu.

### 6.4 Safety considerations

Always handle silver nitrate solutions using gloves, a laboratory coat, and eye protection. Even with the protective enclosure installed, the device must be operated on a dry, stable, and the electrochemical cell should be placed beside the instrument rather than above it to minimize the risk of accidental liquid contact with the electronics. The cell should be used with secondary containment whenever possible.

USB power must be disconnected before preparing the electrolyte, filling the cell, inserting or removing electrodes, opening the enclosure, or modifying any electrical connection. Before reconnecting power, verify that the work area, the exterior of the cell, the connection leads, and the enclosure surfaces are dry, and confirm that all electrical connections are secure. If a spill or splash occurs, disconnect power immediately and inspect the system before further use. The instrument should not be operated unattended.

The platform is intended for low-voltage pulsed constant-current electrodeposition and should not be used for high-voltage anodization, high-current loads beyond the validated operating range, flammable solvent vapors, or unknown electrochemical cells. Because the MOSFET operates in linear mode, any increase in current or modification of the power stage requires evaluation of MOSFET power dissipation, shunt-resistor power rating, voltage compliance, and thermal management.

## 7 Validation and characterization

### 7.1 Current-output validation

The output current was evaluated using a test LED load before connecting the electrochemical cell. A UNI-T UT117C digital multimeter was connected in series with the LED load and the current-control path. Measurements were performed using the 6.0000 A DC-current range, which provides a resolution of 0.1 mA. According to the manufacturer, the DC-current accuracy for this range is ±(0.5% of the reading + 10 counts), corresponding to an expected measurement accuracy of approximately ± 1.00 to ± 1.20 mA over the measured range of 0.60–40.10 mA. Each programmed current was measured five times after the displayed value had stabilized. All five readings were identical at the displayed resolution; therefore, Table 11 reports the common indicated value rather than an arithmetic mean and standard deviation.

**Table 11:** Programmed and multimeter-indicated current values obtained with the Arduino-based pulsed constant-current platform using a test LED load. The UT117C multimeter was connected in series and operated on the 6.0000 A DC-current range.

Programmed current (mA)	Multimeter-indicated current (mA)	Absolute difference (mA)	Relative difference (%)
0.5	0.6	0.1	20.0
1.0	1.1	0.1	10.0
2.0	2.1	0.1	5.0
3.0	3.1	0.1	3.3
4.0	4.1	0.1	2.5
5.0	5.2	0.2	4.0
6.0	6.3	0.3	5.0
7.0	7.3	0.3	4.3
8.0	8.1	0.1	1.3
9.0	9.1	0.1	1.1
10.0	10.1	0.1	1.0
15.0	15.1	0.1	0.7
20.0	20.6	0.6	3.0
25.0	25.2	0.2	0.8
30.0	30.3	0.3	1.0
35.0	35.8	0.8	2.3
40.0	40.1	0.1	0.3

**Fig. 15:**
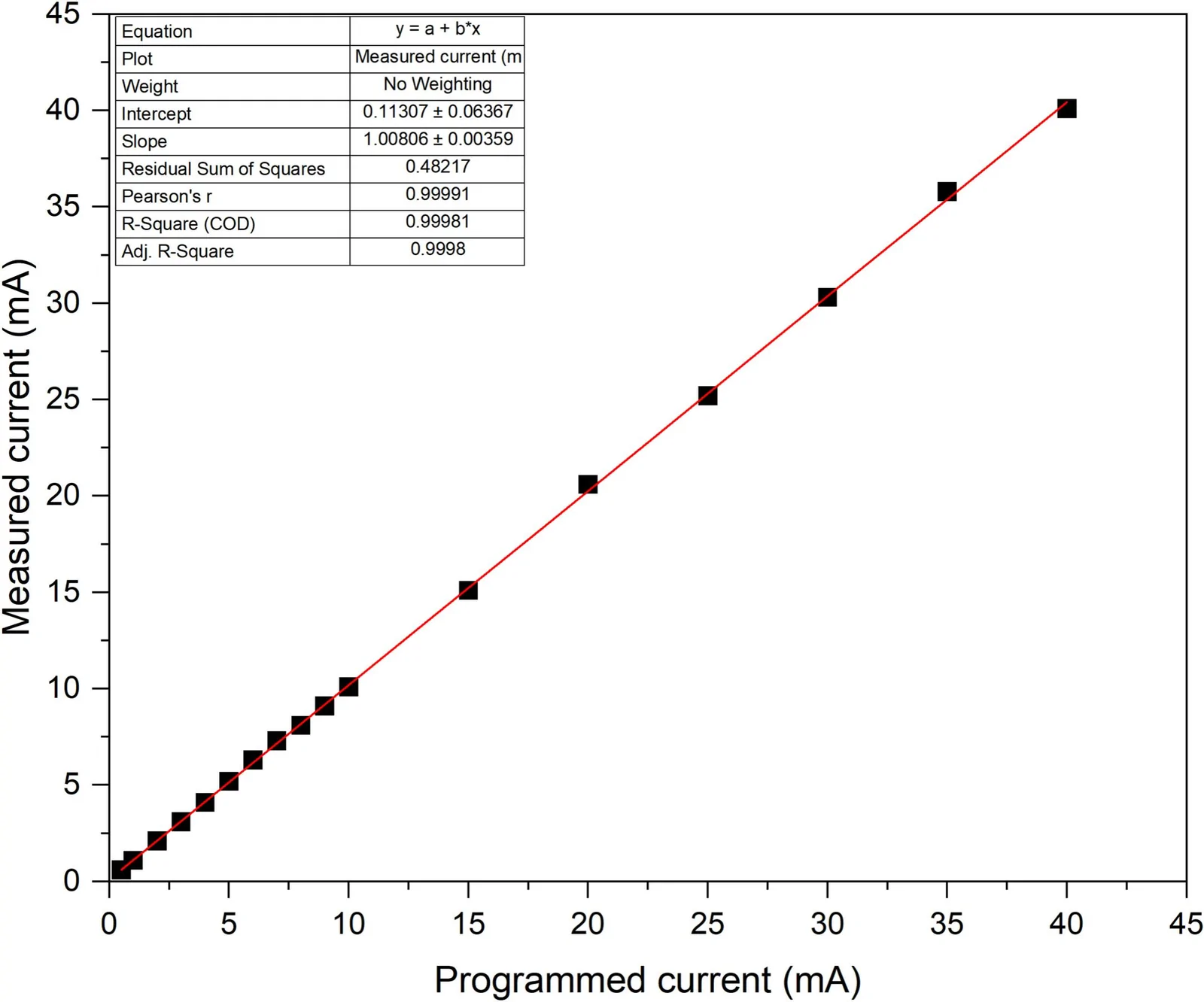
Multimeter-indicated current as a function of programmed current for the Arduino-based pulsed constant-current platform. The linear fit illustrates the highly linear relationship over the evaluated 0.50–40.00 mA range.

Programmed currents ranging from 0.50 to 40.00 mA showed a highly linear relationship with the multimeter-indicated current, with R^2^ = 0.9998. Fig. 15 presents the multimeter-indicated current as a function of the programmed current and the corresponding linear fit. The largest observed difference between the programmed and indicated values was 0.80 mA at the 35.00 mA setpoint, while the mean absolute difference and root-mean-square difference were 0.22 and 0.29 mA, respectively. However, these differences are smaller than the manufacturer-specified accuracy of the reference multimeter and should therefore not be interpreted as an independent determination of the absolute accuracy of the current source. Instead, the results demonstrate linear current response and agreement between programmed and indicated current within the measurement capability of the UT117C. Table 11 provides the programmed and multimeter-indicated current values together with their absolute and relative differences.

### 7.2 Pulse timing and OFF-state verification

**Fig. 16:**
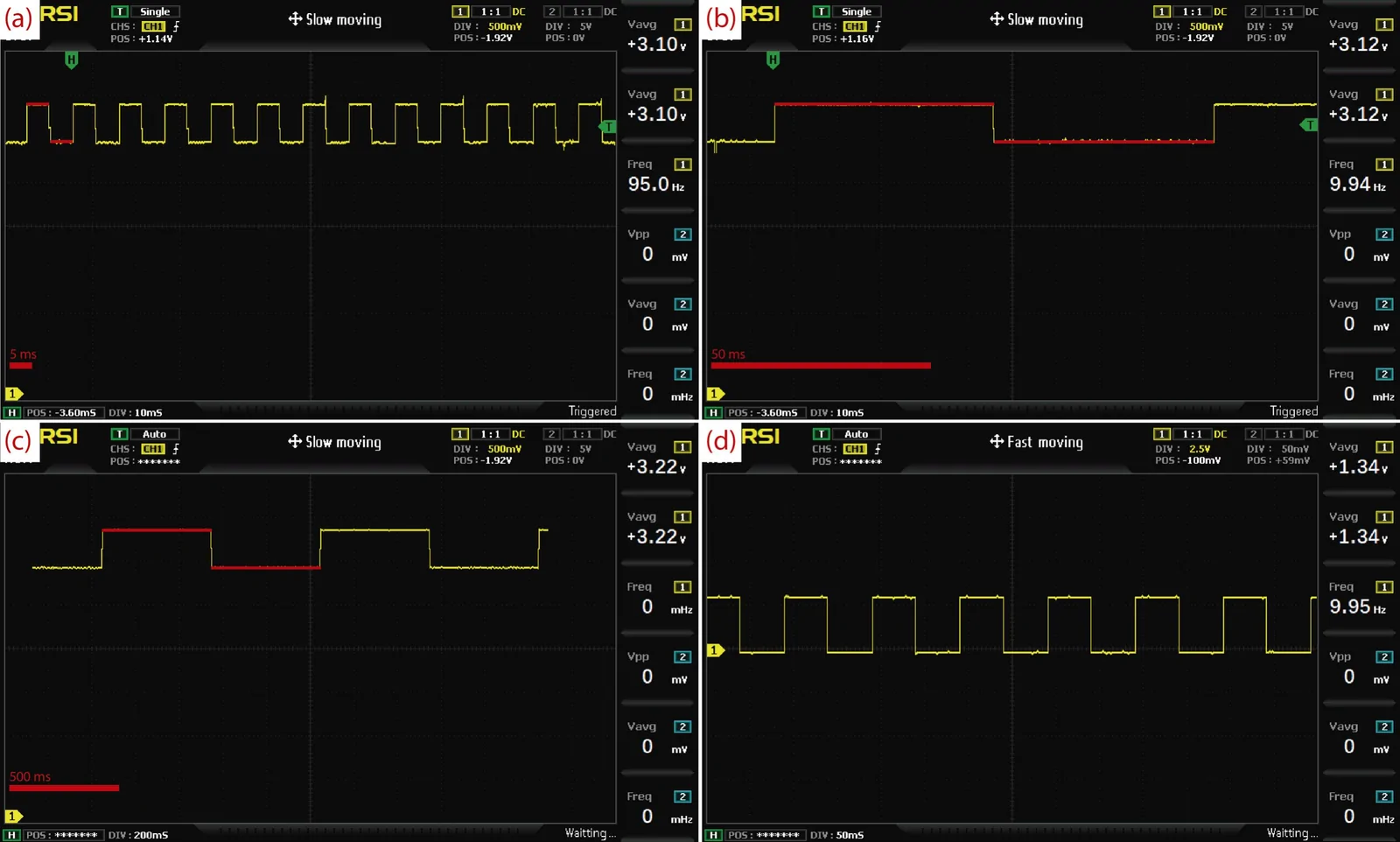
Oscilloscope verification of pulse timing and MOSFET gate switching. (a-c) Square-wave signals recorded for programmed ON/OFF times of 5 ms/5 ms, 50 ms/50 ms, and 500 ms/500 ms, respectively. (d) Gate waveform of the IRLZ34N MOSFET, showing ON/OFF switching and return to the low state during the OFF interval.

**Fig. 17:**
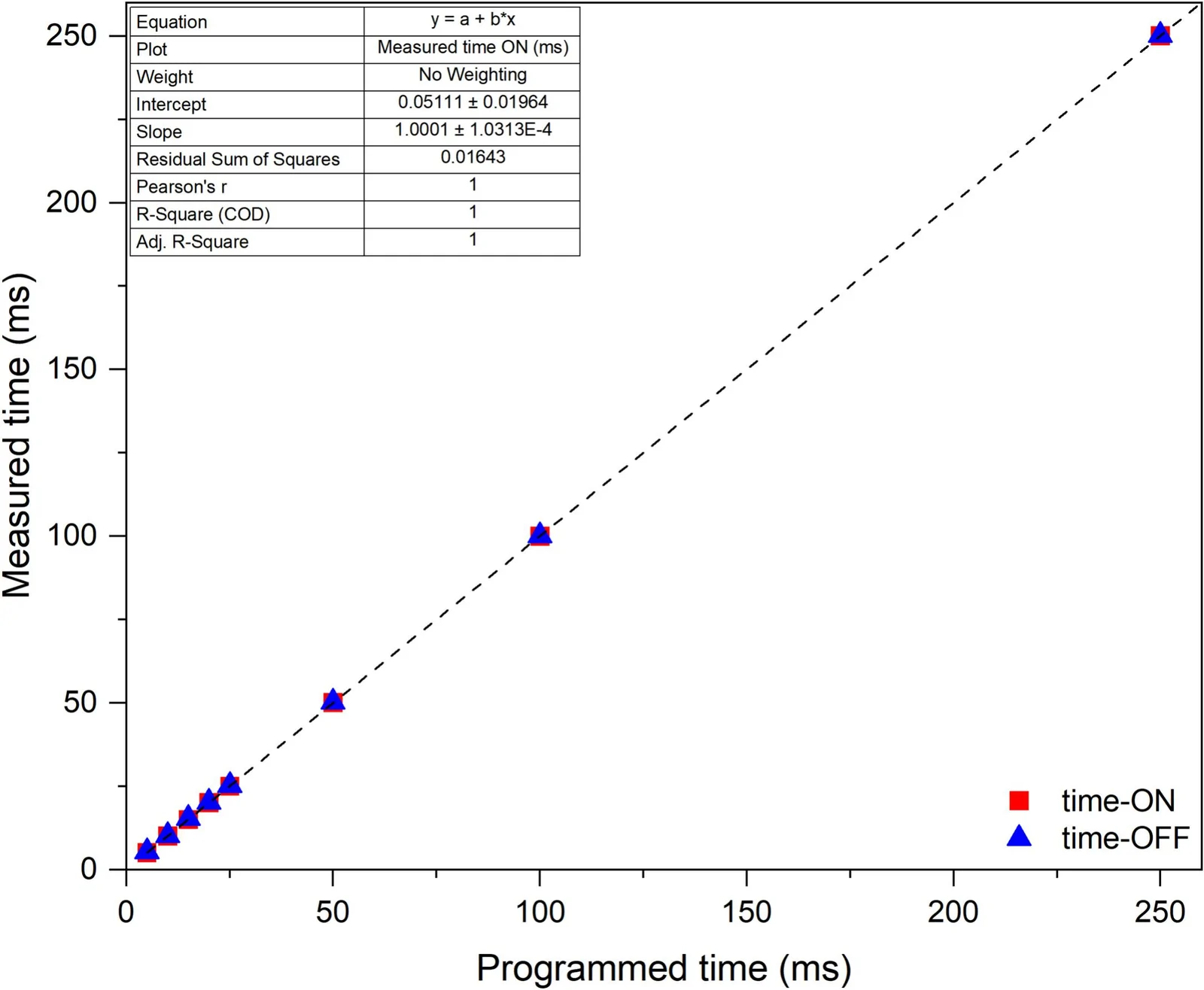
Programmed versus measured ON/OFF pulse durations from 5 to 500 ms. The dashed line indicates the ideal 1:1 response.

Pulse timing was verified using oscilloscope monitoring under representative square-wave pulse conditions. As shown in Fig. 16(a)–(c), the platform generated stable and well-defined pulse signals for programmed ON/OFF times of 5 ms/5 ms, 50 ms/50 ms, and 500 ms/500 ms, respectively. These conditions were selected to evaluate short, intermediate, and long timing intervals within the operational range required for low-current pulsed electrodeposition. The 50 ms condition is particularly relevant because it corresponds to the pulse duration used in representative Ag electrodeposition protocols for TiO_2_/Ag SERS substrate fabrication. The measured ON and OFF pulse durations closely matched the programmed values over the complete evaluated range. The quantitative timing results are summarized in Table 12, where programmed pulse times from 5 to 500 ms are compared with the measured ON and OFF durations. The ON-time absolute difference remained ≤ 0.10 ms for all evaluated values, while the OFF-time absolute difference remained ≤ 0.45 ms. The highest relative OFF-time error was observed at the shortest programmed interval of 5 ms, where small absolute deviations produce a larger percentage error. For programmed times ≥ 50 ms, the relative difference was ≤ 0.2% for ON time and ≤ 0.6% for OFF time. To further evaluate timing linearity, the measured ON and OFF times were plotted as a function of the programmed time, as shown in Fig. 17. Both ON and OFF datasets closely followed the ideal 1:1 response. The ON-time linear fit yielded a slope of 1.0001 and R^2^ ≈ 1.000, indicating excellent timing linearity. However, as summarized in Table 12, small absolute deviations were still observed, particularly for the OFF time at the shortest programmed interval. The OFF-state behavior was further assessed by monitoring the IRLZ34N gate signal. As shown in Fig. 16(d), repeated ON/OFF switching produced a clear return to the low state during the OFF interval. In the firmware, this condition is enforced by setting the DAC command to zero and activating the 2N2222 gate clamp, which discharges the MOSFET gate and minimizes residual conduction during the relaxation period. Collectively, Fig. 16, Fig. 17, together with Table 12, confirm that the platform delivers reproducible pulse timing and a reliable OFF state for pulsed electrodeposition. A direct waveform comparison with a commercial electrochemical workstation was not performed because such equipment was not available in the laboratory. Consequently, synchronized current-transient acquisition and calculation of the deviation between the two instruments at different stages of the pulse cycle could not be conducted. This remains a relevant future validation step under matched load, current setpoint, pulse timing, oscilloscope bandwidth, and probe conditions.

**Table 12:** Programmed and measured ON/OFF pulse durations obtained from oscilloscope monitoring. Absolute and relative differences are reported for both ON time and OFF time over the evaluated timing range from 5 to 500 ms.

Programmed time (ms)	Measured time-ON (ms)	Absolute difference (ms)	Relative difference (%)	Measured time-OFF (ms)	Absolute difference (ms)	Relative difference (%)
5	5.1	0.1	1.2%	5.5	0.5	8.3%
10	10.1	0.1	1.0%	10.3	0.3	2.9%
15	15.0	0.0	0.0%	15.4	0.4	2.6%
20	20.1	0.1	0.5%	20.3	0.3	1.5%
25	25.0	0.0	0.0%	25.3	0.3	1.2%
50	50.1	0.1	0.2%	50.3	0.3	0.6%
100	100.0	0.0	0.0%	100.2	0.2	0.2%
250	250.1	0.1	0.0%	250.3	0.3	0.1%
500	500.1	0.1	0.0%	500.3	0.3	0.1%

### 7.3 Application-level validation using TiO_2_/Ag SERS substrates

**Fig. 18:**
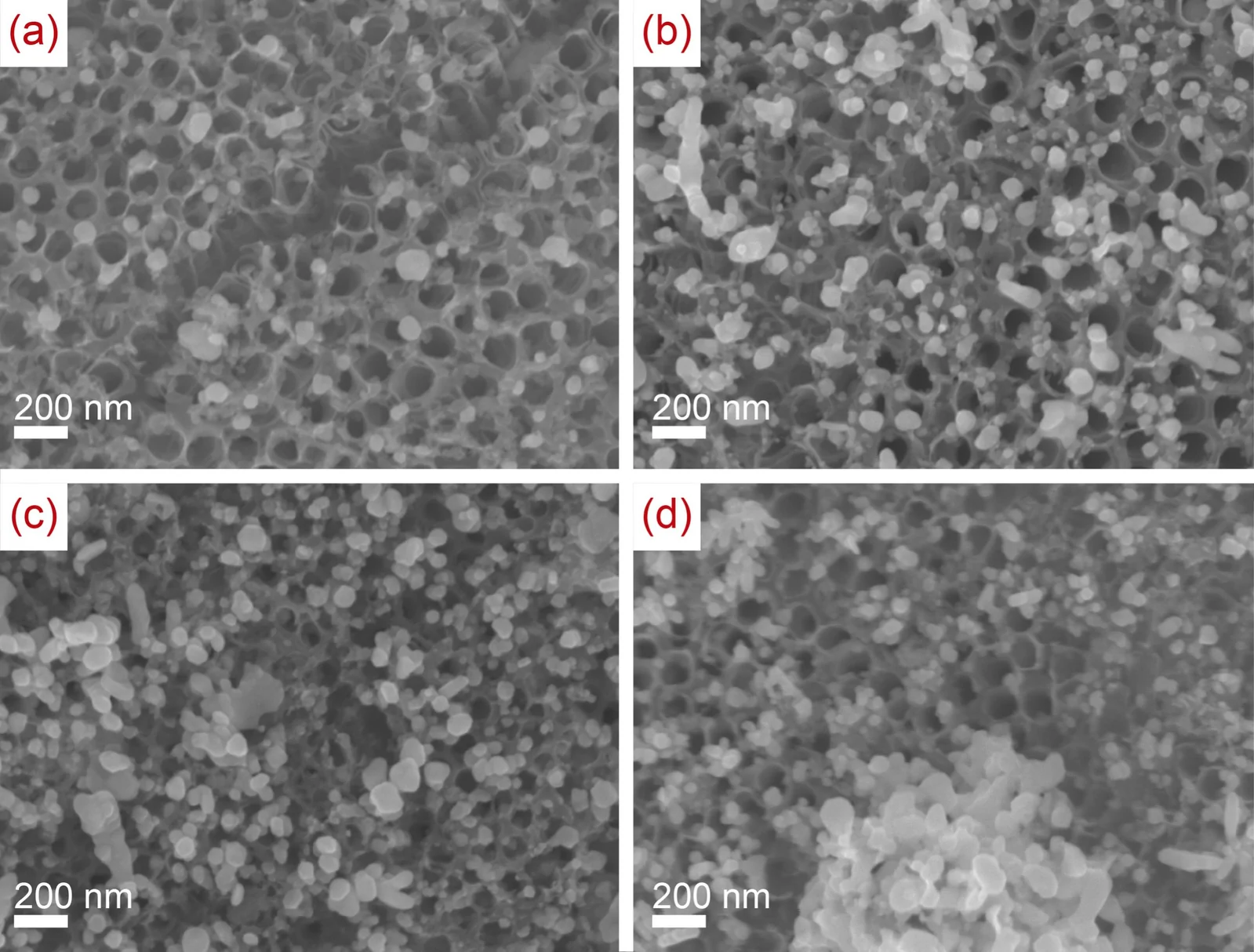
FESEM images of Ag nanoparticles electrodeposited on bamboo-like TiO_2_ nanotubes using 400 pulsed-current cycles at current densities of (a) 4, (b) 5, (c) 6, and (d) 7 mA/cm^2^. Increasing current density produced denser Ag coverage and greater local agglomeration.

**Fig. 19:**
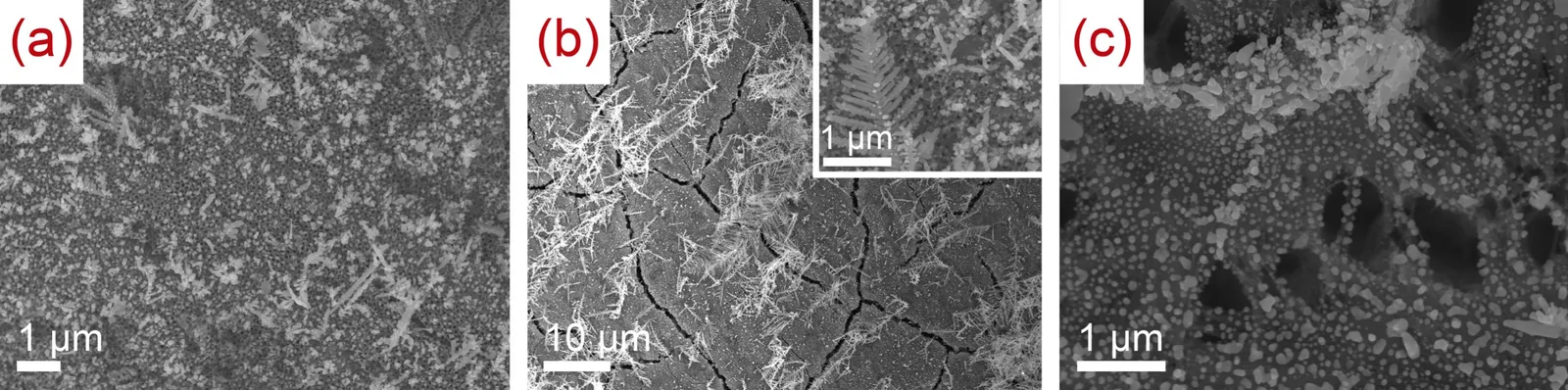
FESEM images showing the effect of TiO_2_ scaffold morphology on Ag electrodeposition at 5 mA/cm^2^ and 400 cycles. (a) Bamboo-like nanotubes prepared at 60/20 V. (b) Cracked bamboo-like nanotubes prepared at 80/10 V, showing preferential dendritic growth along the cracks; the inset shows Ag nanoparticles decorating the nanotubular surface. (c) TiO_2_ nanograss obtained by galvanostatic anodization at 25 mA/cm^2^.

The platform was validated under its intended operating conditions through pulsed-current electrodeposition of Ag nanostructures on anodized TiO_2_ scaffolds [18], [19], [20]. This validation is relevant because SERS activity is highly sensitive to the density, size, spatial distribution, morphology, and interparticle spacing of the deposited metal phase. Thus, the reproducible formation of Ag nanoparticles and dendritic architectures provides application-level evidence that the hardware is suitable for low-voltage electrochemical nanostructure synthesis, beyond electrical characterization alone. The representative micrographs shown in Fig. 18, Fig. 19 were acquired by field-emission scanning electron microscopy (FESEM, JEOL JSM-7600F) using secondary electrons at 15 kV and a working distance of 5.0 mm, following the characterization conditions reported in the authors’ previous studies.

Fig. 18 shows representative FESEM micrographs of Ag nanoparticle morphologies obtained on bamboo-like nanotube arrays prepared in ethylene glycol containing 0.33 wt% NH_4_F using an alternating-voltage sequence of 60/20 V, applied every 2 min for 30 cycles. Ag deposition was performed using 400 pulsed-current cycles while varying the applied current density from 4 to 7 mA/cm^2^. As shown in Fig. 18(a)–(d), increasing current density progressively increased surface coverage. At 4 mA/cm^2^, the particles were more sparsely distributed, whereas higher values produced denser coverage, partial coalescence, and eventually larger local aggregates. The corresponding nanoparticle size and surface coverage values are summarized in Table 13. This behavior is consistent with the expected kinetics of pulsed-current electrodeposition. During the ON interval, Ag^+^ reduction occurs at the oxide/electrolyte interface, whereas the OFF period allows partial ionic redistribution near the electrode surface. Increasing the applied current density raises the instantaneous reduction rate during each pulse, accelerating nucleation and growth. However, when the deposition rate becomes too high, particle coalescence and agglomeration are favored, reducing the population of well-defined nanogaps that act as electromagnetic hotspots. This interpretation agrees with the authors’ previous AgNP optimization study, where current density, pulse duration, and cycle number had to be balanced to maximize SERS performance; excessive metal loading produced overgrowth and reduced enhancement efficiency [20].

**Table 13:** Morphological parameters of Ag nanoparticles electrodeposited on bamboo-like TiO_2_ nanotubes at different current densities using 400 pulsed-current cycles. Nanoparticle diameter, aggregate size, and surface coverage were obtained from SEM image analysis.

Current density (mA/cm^2^)	Ag feature size (nm)	Surface coverage (%)	Morphological observation
4	58 ± 15	18	Sparse AgNP distribution
5	63 ± 10	42	Dense and well-distributed AgNPs
6	71 ± 17	53	Increased coverage and partial coalescence
7	62 ± 21 800 ± 300 (aggregates)	61	Pronounced agglomeration with small AgNPs

The FESEM micrographs in Fig. 19 illustrate the complementary effect of the underlying oxide morphology. In all cases, Ag deposition was carried out at 5 mA/cm^2^ for 400 cycles, but the resulting structures differed markedly depending on the scaffold. On nanotube arrays prepared using the 60/20 V alternating-voltage sequence, Ag formed mainly nanoparticle-rich deposits with limited branched features, as shown in Fig. 19(a). When the anodization sequence was changed to 80/10 V, the oxide layer developed a cracked surface, and Ag dendrites grew preferentially along those regions, Fig. 19(b). The inset in Fig. 19(b) also shows Ag nanoparticles decorating the nanotubular surface, indicating that dendritic growth along cracks occurred together with nanoparticle deposition over the surrounding oxide. This behavior is consistent with the mechanism reported previously, where self-formed cracks and axial segmentation promote localized electric-field concentration, preferential nucleation, and hierarchical dendritic growth [12]. In contrast, the galvanostatically anodized nanograss substrate shown in Fig. 19(c) produced a mixed nanoparticle/aggregate morphology over a high-surface-area oxide network, in agreement with the substrate-dependent growth behavior reported for galvanostatic TiO_2_/Ag systems [11].

Together, Fig. 18, Fig. 19 demonstrate that the proposed hardware can generate distinct Ag morphologies, including nanoparticles, aggregates, and dendritic structures, by combining programmable pulsed-current conditions with controlled oxide scaffolds. The detailed growth mechanisms, SERS optimization, and analytical performance of these systems have been discussed extensively in previous studies by the authors [18], [19], [20]. In the present article, these results are used as application-level validation of the open-source device, showing that the same low-cost platform can fabricate functional TiO_2_/Ag SERS substrates under realistic synthesis conditions. The corresponding peer-reviewed validation cases are summarized in Table 14.

**Table 14:** Comparative SERS performance of Ag-decorated TiO_2_-based and related metallic SERS substrates reported in the literature. AEF: analytical enhancement factor; MB: methylene blue; R6G: rhodamine 6G; MG: malachite green.

Substrate composition	Synthesis method	Analyte	AEF	Detection level (M)	Ref.
AgNWs	Drop-casting Method / Hydrothermal Method	MB	2 × 10^12^	1 × 10^–16^	[21]
AgNPs / TiO_2_ nanotubes	DC Magnetron Sputtering / Constant Voltage Anodization	R6G	−-	1 × 10^–14^	[22]
AgNPs / TiO_2_ nanotubes and nanograss	Constant Voltage Anodization / DC Magnetron Sputtering	MB	1 × 10^5^	1 × 10^–12^	[23]
AgNPs / TiO_2_ nanorods	Chemistry Method	MG	4 × 10^5^	1 × 10^–12^	[24]
AgNPs / TiO_2_ nanospheres	Ultraviolet Light-Induced / Commercial Nanospheres	R6G	7 × 10^10^	1 × 10^–12^	[25]
AgAuNPs	Pulsed laser ablation	MB	1 × 10^8^	1 × 10^–12^	[26]
AuNPs / Cu_2_O microspheres	Photochemical deposition / Polyol method	MB	−-	1 × 10^–12^	[27]
AgNP / Aluminum	Drop-drying Method	MB	2 × 10^9^	5 × 10^–12^	[28]
AgDs / BTNTs	Pulsed Current Electrodeposition / AV Anodization	MB	3 × 10^7^	1 × 10^–11^	[19]
AgDs / TNS	Pulsed Current Electrodeposition / Constant Current Anodization	MB	7 × 10^7^	1 × 10^–11^	[18]
AgTNP@TiO_2_@Ag core-satellite	Chemical Method	MB	−-	1 × 10^–10^	[29]
AgNPs / CoNi	Electrostatic self-assembly	MB	−-	1 × 10^–10^	[30]
AgNPs / TiO_2_ nanosheet	Hydrothermal Method / DC Magnetron Sputtering	R6G	1 × 10^5^	8 × 10^–9^	[31]
AgNPs / TiO_2_ nanotubes	Thermal Evaporation / Constant Voltage Anodization	MB	2 × 10^5^	1 × 10^–8^	[32]
Ag@mTiO_2_ NPs	Hydrothermal process / Chemical Method	MB	−-	1 × 10^–8^	[33]
AgNPs / TiO_2_ nanotubes	Arc Ion Plating Method / DC Magnetron Sputtering	R6G	1 × 10^9^	1 × 10^–8^	[34]
AgNPs / TiO_2_	Ultraviolet Light-Induced / Sol-Gel	R6G	−-	1 × 10^–8^	[35]
AgNPs / BTNTs	Alternating Voltage Anodization / Pulsed Current Electrodeposition	MB	1 × 10^6^	1 × 10^–9^	[20]
AgNDs / TiO_2_ nanotubes	Constant Current Electrodeposition / Constant Voltage Anodization	MB	−-	1 × 10^–^5	[36]

To place the application-level validation in context, Table 14 compares the SERS performance of the TiO_2_/Ag substrates fabricated with the present platform, highlighted in red, against related Ag-decorated TiO_2_-based and metallic substrates reported in the literature. The comparison includes a broad range of synthesis routes, such as electrodeposition, sputtering, thermal evaporation, arc ion plating, hydrothermal synthesis, chemical methods, light-induced deposition, and laser ablation. Several of these approaches can produce highly sensitive SERS substrates; however, they often require specialized instrumentation, vacuum systems, multi-step processing, commercial nanomaterials, or larger reagent and equipment demands. In contrast, the present system uses an open-source, low-cost pulsed-current circuit to control Ag electrodeposition under ambient conditions. Because the compared studies were not performed under identical experimental conditions, Table 14 is intended as a literature-based benchmark of substrate performance rather than as a direct comparison between the proposed circuit and commercial electrochemical instrumentation. Within this scope, the morphologies produced with the proposed hardware show analytical performance within the range reported for established SERS substrates.

The results in Table 14 show that the highest reported AEFs and lowest detection levels are generally associated with architectures designed to promote strong plasmonic coupling, including Ag nanowires, Ag-decorated nanospheres, bimetallic nanoparticles, and other densely coupled metallic structures. Such morphologies can provide elongated features, sharp edges, and numerous interparticle junctions, thereby increasing hotspot density and electromagnetic enhancement. The Ag dendrite substrates fabricated with the present platform yielded AEFs of 3 × 10^7^ and 7 × 10^7^, placing them above several nanoparticle-based TiO_2_ substrates but below the highest-performing nanowire, nanosphere, and bimetallic systems included in the comparison. Their performance is consistent with the presence of branched structures, sharp tips, and interbranch junctions. The AgNP/BTNT substrate yielded a lower AEF of 1 × 10^6^, which may be related to its more rounded particle morphology and to the balance between Ag surface coverage and particle coalescence: insufficient coverage limits nanogap formation, whereas excessive deposition merges adjacent particles and reduces the number of well-defined interparticle junctions. Overall, the range of results indicates that SERS performance depends on metal morphology, hotspot density, surface coverage, interparticle spacing, scaffold geometry, metal composition, and analyte-specific measurement conditions, rather than on the deposition method alone.

### 7.4 Capabilities and limitations

#### 7.4.1 Demonstrated capabilities

• Programmable pulsed current output from 0.50 to 40.00 mA.
• Mean absolute difference of 0.22 mA between programmed and multimeter-indicated current over the evaluated range.
• Maximum observed programmed-to-indicated current difference of 0.80 mA.
• Highly linear relationship between programmed and multimeter-indicated current, with R^2^ = 0.9998.
• Firmware-defined ON/OFF pulse timing.
• Standalone operation using LCD and push-buttons.
• Active hard-OFF gate clamping during relaxation periods.
• Successful use of Ag nanoparticles and Ag dendrite electrodeposition on anodized TiO_2_ substrates.
• Compatibility with low-voltage aqueous Ag electrodeposition protocols.

#### 7.4.2 Limitations and future improvements

• The prototype is limited by the Arduino 5 V supply.
• It is not intended for high-voltage anodization or high-current electrodeposition.
• It does not provide potentiostatic control.
• It does not include real-time current logging in the present version.
• The platform controls absolute current in mA, not current density; current density must be calculated externally from the exposed electrode area.
• Voltage compliance depends on the cell voltage, shunt voltage, and MOSFET operating point.
• Replicated units should be verified before use because shunt tolerance, wiring, and supply voltage can affect the delivered current.
• Scaling to higher currents requires redesigning the power stage and evaluating MOSFET heating.
• The present implementation was assembled at the prototype level; a dedicated PCB would improve electrical robustness, reduce wiring variability, and facilitate reproducibility.

### 7.5 Conclusion

A low-cost Arduino-based pulsed constant-current platform was developed for electrochemical nanostructure synthesis under low-voltage conditions. The system combines a DAC-defined current command, an operational-amplifier–MOSFET feedback loop, a 10 Ω shunt resistor, and an active 2N2222 gate clamp to generate programmable current pulses with a well-defined OFF state. ON time, OFF time, cycle number, and current can be configured directly from a 20 × 4 LCD interface, without requiring an external computer during operation. Electrical validation showed a highly linear relationship between programmed and multimeter-indicated current over the evaluated range of 0.50–40.00 mA, with a maximum observed difference of 0.80 mA and R^2^ = 0.9998. The platform was further validated through Ag electrodeposition protocols used to fabricate TiO_2_/Ag SERS substrates, including Ag nanoparticles and 3D Ag dendrites, whose analytical performance was within the range reported for established SERS architectures. These results demonstrate that a simple, inexpensive, and open-source circuit can provide sufficient current regulation, pulse timing, and OFF-state control for reproducible low-current pulsed electrodeposition. Near-term development will prioritize a dedicated PCB, external voltage-compliance control, onboard current sensing, data logging, Bluetooth or USB communication, and improved electrical isolation.

**Declaration of generative AI and AI-assisted technologies in the manuscript preparation process**.

During the preparation of this work, the authors used Grammarly for grammar and spelling correction. The authors reviewed and edited the output as needed and take full responsibility for the content of the published article.

Specifications table.

**Table:** 

Hardware name	Low-Cost Arduino-Based Pulsed Constant-Current Platform for Electrochemical Nanostructure Synthesis
Subject area	• Engineering and materials science
Hardware type	• Mechanical engineering and materials science
Closest commercial analog	Programmable galvanostats/potentiostats with pulsed-current capability*.*
Open source license	Hardware: CERN-OHL-P-2.0Software: MIT LicenseDocumentation / Illustrations / Drawings / Figures: CC BY 4.0
Cost of hardware	< $30.00
Source file repository	Zenodo, https://doi.org/10.5281/zenodo.20750169

### CRediT authorship contribution statement

**Marcos Luna Cervantes:** Writing – review & editing, Writing – original draft, Visualization, Validation, Software, Investigation, Data curation, Conceptualization. **Leandro García González:** Validation, Supervision, Data curation. **Julián Hernández Torres:** Validation, Supervision, Investigation, Data curation. **Erick Octavio Santos Santiago:** Validation, Methodology, Investigation, Data curation. **José Luis Zamora Navarro:** Validation, Methodology, Investigation, Data curation. **Diana Jiménez Girón:** Validation, Methodology, Investigation, Data curation. **Daniel de Jesús Araujo Pérez:** Writing – review & editing, Writing – original draft, Validation, Software, Data curation. **Bertin Santaella González:** Validation, Methodology, Investigation, Data curation. **Mario Alberto Díaz Solís:** Validation, Methodology, Investigation, Data curation. **Pablo Thomas Dupont:** Validation, Methodology, Investigation, Data curation. **Irma Yadira Izaguirre Hernández:** Validation, Methodology, Investigation, Data curation. **Antonio de Jesús García Chávez:** Writing – review & editing, Writing – original draft, Validation, Software, Data curation.

## Declaration of competing interest

The authors declare that they have no known competing financial interests or personal relationships that could have appeared to influence the work reported in this paper.

## References

[1] Bicelli L., Bozzini B., Mele C., D'Urzo L. (2008). A review of nanostructural aspects of metal electrodeposition. Int. J. Electrochem. Sci..

[2] Luo G., Yuan Y., Li D., Li N., Yuan G. (2022). Current transition of nucleation and growth under diffusion-controlled electrocrystallization: a brief review. Coatings.

[3] Daryadel S., Behroozfar A., Morsali S., Moreno S., Baniasadi M., Bykova J., Bernal R., Minary-Jolandan M. (2018). Localized pulsed electrodeposition process for three-dimensional printing of nanotwinned metallic nanostructures. Nano Lett..

[4] Paul S. (2015). Nanomaterials synthesis by electrodeposition techniques for high-energetic electrodes in fuel cell. Nanomaterials and Energy.

[5] Shiohara A., Wang Y., Liz-Marzán L. (2014). Recent approaches toward creation of hot spots for SERS detection. J Photochem Photobiol C: Photochem Rev.

[6] Hardy M., Goldberg Oppenheimer P. (2024). “When is a hotspot a good nanospot”: review of analytical and hotspot-dominated surface enhanced Raman spectroscopy nanoplatforms. Nanoscale.

[7] Zhou X., Liu S., Xiang H., Li X., Wang C., Wu Y., Li G. (2025). Three-dimensional SERS substrates: architectures, hot spot engineering, and biosensing applications. Biosensors.

[8] Lai Y., Zhuang H., Xie K., Gong D., Tang Y., Sun L., Lin C., Chen Z. (2010). Fabrication of uniform Ag/TiO_2_ nanotube array structures with enhanced photoelectrochemical performance. New J. Chem..

[9] Xie K., Sun L., Wang C., Lai Y., Wang M., Chen H., Lin C. (2010). Photoelectrocatalytic properties of Ag nanoparticles loaded TiO_2_ nanotube arrays prepared by pulse current deposition. Electrochim. Acta.

[10] Guan D., Wang Y. (2013). Electrodeposition of Ag nanoparticles onto bamboo-type TiO_2_ nanotube arrays to improve their lithium-ion intercalation performance. Ionics.

[11] von Zuben T., Salles A., Bonacin J. (2024). Low-cost open-source potentiostats: a comprehensive review of DIY solutions and fundamental concepts of electronics and its integration with electrochemistry. Electrochim. Acta.

[12] Caux M., Achit A., Var K., Boitel-Aullen G., Rose D., Aubouy A., Argentieri S., Campagnolo R., Maisonhaute E. (2022). PassStat, a simple but fast, precise and versatile open source potentiostat. HardwareX.

[13] Irving P., Cecil R., Yates M. (2021). MYSTAT: a compact potentiostat/galvanostat for general electrochemistry measurements. HardwareX.

[14] Guerrero-Felix J., Lopez-Miras J., Rodriguez-Valverde M., Moraila-Martinez C., Fernandez-Rodriguez M. (2023). Automation of an atomic force microscope via Arduino. HardwareX.

[15] Shnier A., Otieno F., Billing C., Wamwangi D., Billing D. (2023). Robust Arduino controlled spin coater using a novel and simple gravity chuck design. HardwareX.

[16] Le A., Nguyen Q., Pham B., Cao T., Vo T., Huynh K., Ha H. (2023). SALAD: Syringe-based Arduino-operated Low-cost Antibody Dispenser. HardwareX.

[17] Fischer M., Fischer M. (2024). Cost-effective, open-source light shutters with Arduino control. HardwareX.

[18] Luna-Cervantes M., García-Ramírez J., Hernández-Torres J., Zamora-Peredo L. (2025). Current density optimization for SERS substrates using galvanostatically anodized TiO_2_ nanostructures decorated with pulsed-electrodeposited silver dendrites. Materials.

[19] Luna-Cervantes M., García-Ramírez J., Hernández-Torres J., Zamora-Peredo L. (2025). Pulsed-electrodeposited 3D silver dendrites on bamboo-like TiO_2_ nanotubes for ultrasensitive SERS detection. J. Electrochem. Soc..

[20] M. Luna Cervantes, I. García-Ramírez, E.O. Santos Santiago, D. Jiménez Girón, J.L. Zamora Navarro, A. García Chavez, L. García-González, A. Báez-Rodríguez, J. Hernández Torres, L. Zamora-Peredo, Pulsed current electrodeposition of Ag nanoparticles on bamboo-like TiO_2_ nanotubes for surface-enhanced Raman scattering substrates, ACS Omega 10 (50) (2025) 62257-62267, https://doi.org/10.1021/acsomega.5c10021. 10.1021/acsomega.5c10021 PMC12750385 41476500

[21] I. Horta, N. Azevedo Neto, L. Terumi Kito, F. Miranda, G. Thim, A. Pereia, R. Pessoa, Ultra-trace monitoring of methylene blue degradation via AgNW-based SERS: toward sustainable advanced oxidation water treatment, Sustainability 17 (10) (2025) 4448, https://doi.org/10.3390/su17104448.

[22] Lamberti A., Virga A., Chiado A., Chiodoni A., Bejtka K., Rivolo P., Giorgis F. (2015). Ultrasensitive Ag-coated TiO_2_ nanotube arrays for flexible SERS-based optofluidic devices. J. Mater. Chem. C.

[23] Kadir M., Nemkayeva R., Baigarinova G., Alpysbayeva B., Assembayeva A., Smirnov V. (2023). SERS-active substrates based on Ag-coated TiO_2_ nanotubes and nanograss. Physica E.

[24] En-Zhong T., Peng-Gang Y., Ting-ting Y., Hua W., Lin G. (2012). Three dimensional design of large-scale TiO_2_ nanorods scaffold decorated by silver nanoparticles as SERS sensor for ultrasensitive malachite green detection. ACS Appl. Mater. Interfaces.

[25] Jiang M., Wang Z., Zhang J. (2022). TiO_2_/AgNPs SERS substrate for the detection of multi-molecules with a self-cleaning and high enhancement factor using the UV-induced method. Optical Materials Express.

[26] Kadavath J., Krishnan B., Cienfuegos R., Sepúlvedda S., Garcia-Gomez N., Avellaneda D., Shaji S. (2025). Ultrasensitive and stable SERS sensors by incorporating noble metal/bimetallic nanoparticles produced by laser ablation in liquid. Microchem. J..

[27] Elumalai K., Tzyy-Jiann W., Yu-Hsu C. (2022). Ultrasensitive SERS substrates based on Au nanoparticles photo-decorated on Cu_2_O microspheres for the detection of rhodamine B and methylene blue. Appl. Surf. Sci..

[28] Mai Quand D., Nguyen Ha A., Nguyen Xuan Q., Ngo Xuan D., Doan Quang T., Tran Quang H., Anh-Tuan L. (2022). Ultrasensitive detection of methylene blue using an electrochemically synthesized SERS sensor based on gold and silver nanoparticles: roles of composition and purity on sensing performance and reliability. J. Electron. Mater..

[29] Cheng H., Luo K., Wen X., Yang J., Li J. (2024). AgTNP@TiO_2_@Ag core-satellite composites for sensitive sensing and in situ monitoring photodegradation of organic dyes by portable Raman spectrometer. Spectrochim. Acta A Mol. Biomol. Spectrosc..

[30] Ren S., Fu J., Liu G., Zhang H., Wang B., Yu J. (2024). Ultrasensitive detection of methylene blue by surface-enhanced Raman scattering (SERS) with Ag nanoparticle-decorated magnetic CoNi layered double hydroxides. Anal. Methods.

[31] Yang L., Wang W., Jiang H., Zhang Q., Shan H., Zhang M., Zhu K., Lv J., He G., Sun Z. (2017). Improved SERS performance of single-crystalline TiO_2_ nanosheet arrays with coexposed 001 and 101 facets decorated with Ag nanoparticles. Sens. Actuators B.

[32] Pisarek M., Krawczyk M., Hołdynski M., Ambroziak R., Bienkowski K., Roguska A., Krajczewski J., Kudelski A. (2023). Hybrid nanostructures based on TiO_2_ nanotubes with Ag, Au, or bimetallic Au-Ag deposits for surface-enhanced Raman scattering (SERS) applications. J. Phys. Chem. C.

[33] Min Y., Song G., Zhou L., Wang X., Liu P., Li J. (2022). Silver@mesoporous anatase TiO_2_ core-shell nanoparticles and their application in photocatalysis and SERS sensing. Coatings.

[34] Hsing-Yu W., Hung-Chun L., Yung-Hsien L., Kai-Lin C., Yu-Hsun W., Yung-Shin S., Jin-Cherng H. (2022). Highly sensitive, robust, and recyclable TiO_2_/AgNP substrate. Molecules.

[35] Huaizhou J., Qipeng L., Shangzhong J., Haiquan D., Hongzhi G., Xingdan C., Yanqiu Z. (2017). The improvements on TiO_2_ catalyzed AgNPs based SERS substrate and detection methods. Vibrational Spectrocopy.

[36] M. Pérez Pérez, E. Cornejo-Zameza, A. Maytorena-Sánchez, L. Zamora-Peredo, J. Hernández-Torres, L. García-González, C. Fereira-Palma, O. Velázquez-Camilo, SERS impact of an electrodeposited nanostructured surface with Ag nanostructures, MRS Advances 9 (23) (2024) 1838-1842, https://doi.org/10.1557/s43580-024-00984-0.

